# Molecular Mechanisms of Antiproliferative and Apoptosis Activity by 1,5-Bis(4-Hydroxy-3-Methoxyphenyl)1,4-Pentadiene-3-one (MS13) on Human Non-Small Cell Lung Cancer Cells

**DOI:** 10.3390/ijms22147424

**Published:** 2021-07-10

**Authors:** Wan Nur Baitty Wan Mohd Tajuddin, Faridah Abas, Iekhsan Othman, Rakesh Naidu

**Affiliations:** 1 Jeffrey Cheah School of Medicine and Health Sciences, Monash University Malaysia, Jalan Lagoon Selatan, Bandar Sunway 47500, Selangor Darul Ehsan, Malaysia; wan.wanmohdtajuddin@monash.edu (W.N.B.W.M.T.); iekhsan.othman@monash.edu (I.O.); 2 Laboratory of Natural Products, Faculty of Science, Universiti Putra Malaysia, UPM, Serdang 43400, Malaysia; faridah_abas@upm.edu.my; 3 Department of Food Science, Faculty of Food Science and Technology, Universiti Putra Malaysia, UPM, Serdang 43400, Malaysia; 4 Global Asia in the 21s Century Platform, Monash University Malaysia, Jalan Lagoon Selatan, Bandar Sunway 47500, Selangor Darul Ehsan, Malaysia

**Keywords:** lung cancer, curcumin analogue, diarylpentanoid, cytotoxicity, anti-proliferation, apoptosis, gene expression

## Abstract

Diarylpentanoid (DAP), an analog that was structurally modified from a naturally occurring curcumin, has shown to enhance anticancer efficacy compared to its parent compound in various cancers. This study aims to determine the cytotoxicity, antiproliferative, and apoptotic activity of diarylpentanoid MS13 on two subtypes of non-small cell lung cancer (NSCLC) cells: squamous cell carcinoma (NCI-H520) and adenocarcinoma (NCI-H23). Gene expression analysis was performed using Nanostring PanCancer Pathways Panel to determine significant signaling pathways and targeted genes in these treated cells. Cytotoxicity screening revealed that MS13 exhibited greater inhibitory effect in NCI-H520 and NCI-H23 cells compared to curcumin. MS13 induced anti-proliferative activity in both cells in a dose- and time-dependent manner. Morphological analysis revealed that a significant number of MS13-treated cells exhibited apoptosis. A significant increase in caspase-3 activity and decrease in Bcl-2 protein concentration was noted in both MS13-treated cells in a time- and dose-dependent manner. A total of 77 and 47 differential expressed genes (DEGs) were regulated in MS13 treated-NCI-H520 and NCI-H23 cells, respectively. Among the DEGs, 22 were mutually expressed in both NCI-H520 and NCI-H23 cells in response to MS13 treatment. The top DEGs modulated by MS13 in NCI-H520—DUSP4, CDKN1A, GADD45G, NGFR, and EPHA2—and NCI-H23 cells—HGF, MET, COL5A2, MCM7, and GNG4—were highly associated with PI3K, cell cycle-apoptosis, and MAPK signaling pathways. In conclusion, MS13 may induce antiproliferation and apoptosis activity in squamous cell carcinoma and adenocarcinoma of NSCLC cells by modulating DEGs associated with PI3K-AKT, cell cycle-apoptosis, and MAPK pathways. Therefore, our present findings could provide an insight into the anticancer activity of MS13 and merits further investigation as a potential anticancer agent for NSCLC cancer therapy.

## 1. Introduction

Lung cancer is the most commonly diagnosed cancer worldwide with a high mortality rate [1]. Lung cancer is grouped into two main types: non-small cell lung cancer (NSCLC) and small cell lung cancer (SCLC). NSCLC is the most common type of lung cancer, representing approximately 80% of all lung cancer cases compared to SCLC (20%). NSCLC is further classified into three subtypes: squamous cell carcinoma (40%), adenocarcinoma (30%) and large cell carcinoma (10%) [2]. The main risk factor of both NSCLC and SCLC is tobacco smoking, with 80–90% of lung cancer patients have smoking history [3]. To date, the standard treatments for both NSCLC and SCLC patients are surgery, chemotherapy, radiation therapy, or a combination of these treatments [4]. Despite various advanced diagnostic and therapeutic approaches of lung cancer, the overall 5-year survival rate remains poor: 10% to 20% for all stages combined [1]. Additionally, several side effects and toxicities of chemotherapy and radiation therapy have been reported in lung cancer patients [4,5]. Thus, there is a need to identify more effective and non-toxic therapeutic drugs for the treatment of lung cancer patients.

Phytocompounds represent one of the promising therapeutic agents that has been studied extensively in preventing and treating various human diseases with reduced side effects [6]. Curcumin, the main component extracted from *Curcuma longa*, has been well documented to possess numerous therapeutic properties including anti-inflammatory [7], antioxidative [8], antimicrobial [9], and anticancer [10]. Previous in vitro and in vivo studies revealed that curcumin acts as a potential candidate of chemopreventive agent and a novel adjuvant for multiple cancer types including lung cancer [11]. Curcumin has been indicated to exhibit its anticancer effect in lung cancer cells through various mechanisms including inhibition of cell proliferation, metastasis and invasion, induction of apoptosis, and regulation of microRNAs expression. These mechanisms were governed by multiple molecular targets and signaling pathways such as STAT3, EGFR, FOXO3a, TGF-β, eIF2α, COX-2, Bcl-2, PI3KAkt/mTOR, ROS, Fas/FasL, Cdc42, E-cadherin, MMPs, and adiponectin [12]. However, despite its broad therapeutic properties as an anticancer agent, pharmacokinetic profile studies have highlighted the bioavailability limitation of curcumin as it has poor absorption and rapid metabolism [13]. Therefore, numerous chemically modified compounds of curcumin have been synthesized to overcome the curcumin limitations while retaining its efficacy as well as safety profile. 

Diarylpentanoids (DAPs) are curcumin analogs with a 5-carbon chain between their aryl rings that have been demonstrated to have more significant anti-tumorigenic effect compared to curcumin. Multiple experimental studies showed that DAPs exhibited anticancer therapeutic properties through the growth inhibition and induction of apoptosis in human cancer cells. Among the DAPs, GO-Y030, FLLL-11, FLLL-12, HO-3867, EF24, and EF31 demonstrated greater growth inhibitory effect in various human cancer cells compared to its parent compound, curcumin [14]. These compounds have been shown to exert its growth inhibitory effect by mediating a wide range of signaling pathways and molecular targets including NF-κB, PI3K/PTEN/Akt/mTOR, MAPK/ERK pathway, VEGF signaling, cell cycle arrest, and apoptotic pathways [15,16,17]. 

MS13, also known as 1,5-bis(4-hydroxy-3-methoxyphenyl)1,4-pentadiene-3-one, is a mono-ketone derivative that holds α- and β-unsaturated ketone moiety [18]. This compound has been indicated to exhibit greater cytotoxic effect with lower EC_50_ values towards prostate [19], cervical [20], colon [21], glioblastoma, and neuroblastoma [22] cancer cells. Additionally, MS13 demonstrated its anticancer activity through induction of apoptosis in colon [21], glioblastoma, and neuroblastoma [22] cancer cells. To the best of our knowledge, this is the first study to evaluate anticancer properties of 1,5-bis(4-hydroxy-3-methoxyphenyl)1,4-pentadiene-3-one against NSCLC cells and its underlying molecular mechanisms. Therefore, in the present study, we investigated the cytotoxicity, antiproliferative, and apoptotic activity of MS13 in NSCLC squamous cell carcinoma (NCI-H520) and adenocarcinoma (NCI-H23). Furthermore, we investigated the underlying molecular mechanisms in MS13-treated human NSCLC cells by identifying differentially expressed genes (DEGs) and associated signaling pathways using the Nanostring PanCancer Pathways Panel. In this study, we highlighted the signaling pathways that were highly associated with NSCLC including PI3K-AKT, cell cycle-apoptosis, and MAPK pathways.

## 2. Results

### 2.1. Cytotoxic Effects of MS13 on NCI-H520 and NCI-H23 Cell Lines

**Figure 1:**
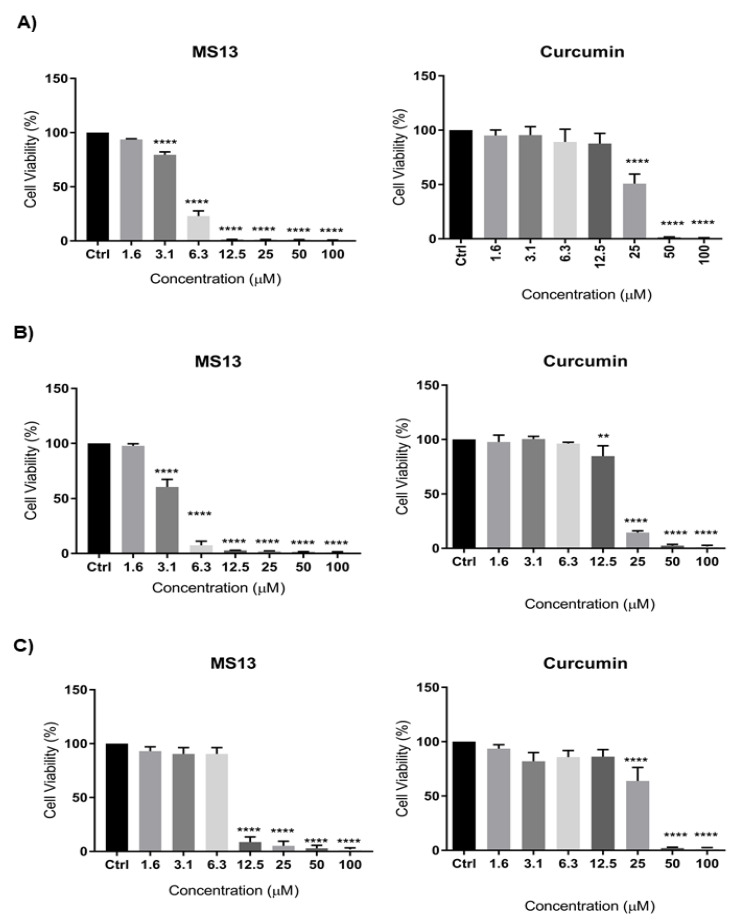
The cytotoxic effects of MS13 and curcumin at different concentrations on (**A**) NCI-H520, (**B**) NCI-H23, and (**C**) MRC9 cell lines compared to untreated sample (Ctrl) at 72 h of incubation. The experiments were performed in triplicates, and results were compared between three independent experiments. The data are presented as means of percentage of cell viability and comparison between data sets were statistically analyzed using ANOVA. Asterisks indicate statistically significant differences between the means of the values obtained with treated versus untreated cells (** *p* < 0.01, and **** *p* < 0.0001).

The dose-dependent cytotoxic effect of MS13 and curcumin on human lung cancer cell lines NCI-H520 and NCI-H23 was determined by evaluating the cell viability using MTT assay at 72 h as depicted in Figure 1. MS13 showed a significant inhibition of growth on NCI-H520 with a significant decrease in cell viability to 79% at 3.1 µM and gradually to 23% at 6.3 µM and less than 5% at 12.5 µM onwards. Similarly, MS13 demonstrated a significant cell inhibitory effect on NCI-H23 at 3.1 µM by decreasing the cell viability to 60% followed by less than 10% at 6.3 µM onwards compared to the control. On the other hand, curcumin demonstrated significant decrease in cell viability on NCI-H520 and NCI-H23 cells at 25 µM (50%) and 12.5 µM (85%), respectively. 

**Figure 2:**
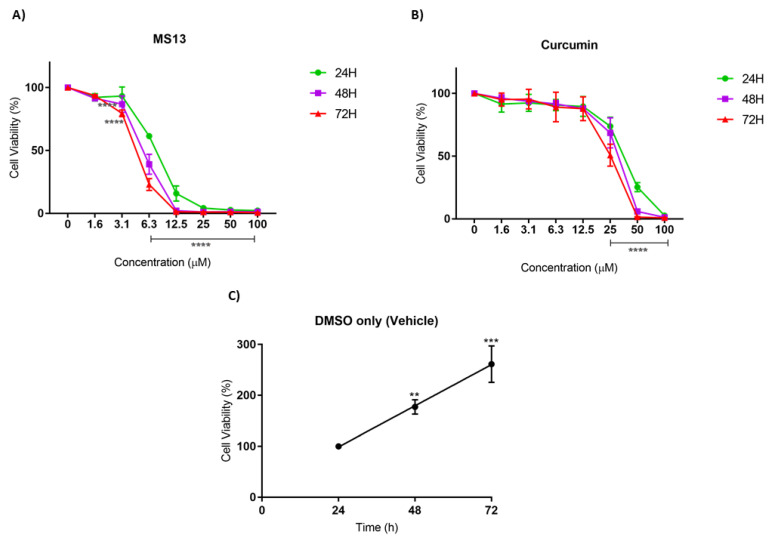
The antiproliferative effects of (**A**) MS13 and (**B**) curcumin on NCI-H520 cell line at 24, 48, and 72 h. The experiments were performed in triplicate and results were compared between three independent experiments. Results are presented as means of percentage of cell viability and comparison between data sets were statistically analyzed using ANOVA. Vehicle-treated controls of DMSO (**C**) were included to assess cell growth in untreated NCI-H520 cells over time. Asterisks indicate statistically significant differences between the means of the values obtained with treated versus untreated cells (** *p* < 0.01, *** *p* < 0.001 and **** *p* < 0.0001).

Table 1 showed the EC_50_ values of MS13 and curcumin on NCI-H520 and NCI-H23 cells obtained from cytotoxicity data. MS13 demonstrated lower EC_50_ value in NCI-H23 cells (3.7 ± 0.4 µM) compared to NCI-H520 cells (4.7 ± 0.1 µM). These results indicate that MS13 possess greater inhibitory effect against NCI-H23 in comparison with NCI-H520. Curcumin showed higher EC_50_ value in both NCI-H520 (25.2 ± 1.7 µM) and NCI-H23 (18.5 ± 0.7 µM) compared to MS13. The cytotoxic effects of MS13 as well as curcumin were further evaluated on the non-cancerous lung fibroblast cell line MRC9 (Figure 2). The results demonstrated that the EC_50_ values of both compounds were higher in MRC9 compared to cancer cell lines, indicating higher dose of MS13 and curcumin was required to decrease MRC9 cell viability by 50%. MS13 and curcumin showed SX values above 100 in NCI-H520 and NCI-H23 cells indicating that the cytotoxic effect of both the compounds is greater towards cancer cells compared to normal cells (Table 1). MS13 exhibited comparably higher SX values in both NCI-H520 and NCI-H23 cells compared to curcumin. 

### 2.2. Antiproliferative Effect of MS13 on NCI-H520 and NCI-H23 Cell Lines

**Figure 3:**
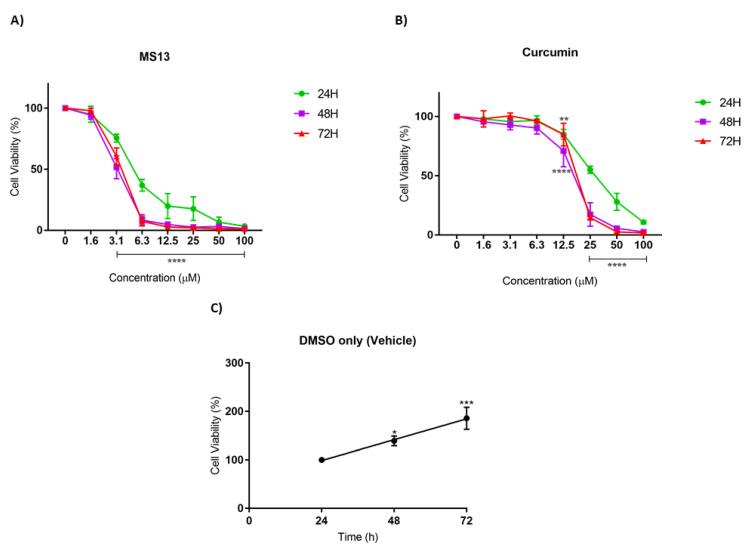
The antiproliferative effects of (**A**) MS13 and (**B**) curcumin on NCI-H23 cell line at 24, 48, and 72 h. The experiments were performed in triplicate and results were compared between three independent experiments. Results are presented as means of percentage of cell viability and comparison between data sets were statistically analyzed using ANOVA. Vehicle-treated controls of DMSO (**C**) were included to assess cell growth in untreated NCI-H520 cells over time. Asterisks indicate statistically significant differences between the means of the values obtained with treated versus untreated cells (* *p* < 0.05, ** *p* < 0.01, *** *p* < 0.001 and **** *p* < 0.0001).

The antiproliferative effect of MS13 and curcumin against NCI-H520 and NCI-H23 cells was assessed by measuring the viability of treated cells at 24, 48, and 72 h. In addition, the cell viability of vehicle-treated (DMSO only) cells was measured to assess the cell proliferation in the absence of MS13 treatment. A significant increase of cell viability was observed from 24 to 72 h in vehicle-treated cells, suggesting that the cell proliferation increases as a function of time (Figure 2C and Figure 3C). However, the proliferation rate of the treated cells was significantly reduced in a dose- and time-dependent manner. 

As depicted in Figure 2A, the MS13 treatment against NCI-H520 cells showed a significant decline in cell viability to 61% at 6.3 µM upon 24 h incubation. At a dosage of 3.1 µM, a significant decrease in cell viability to 86% and 78% at 48 h and 72 h, respectively, was noted. Cell viability of less than 50% was observed upon MS13 treatment at 6.3 µM for 48 and 72 h as well as 12.5 µM for 24 h. The cell viability showed a significant decrease at 6.3 µM following incubation for 48 h (40%) and 72 h (30%). Cell viability of less than 15% was observed following 12.5 µM of MS13 treatment at all time points. Meanwhile, NCI-H520 cell treated with curcumin at 25 µM demonstrated a significant reduction in cell viability to 74%, 69%, and 51% at 24, 48, and 72 h, respectively (Figure 2B). At a dosage of 50 µM, curcumin significantly decreased the cell viability of NCI-H520 cells to less than 50% at all time points.

As for NCI-H23 cells, the MS13 treatment at 3.1 µM significantly reduced cell viability to 75%, 52%, and 60% at 24 h, 48 h, and 72 h, respectively (Figure 3A). Upon MS13 treatment at 6.3 µM, cell viability less than 50% was observed at all time points. At a dosage of 12.5 µM onwards, MS13 treatment significantly reduced the cell viability to less than 20% at 24 h and 3–5 % at 48 h and 72 h. On the other hand, curcumin treatment at 25 µM onwards against NCI-H23 cells exhibited a significant decline to 55% after 24 h of incubation (Figure 3B). At a dosage of 12.5 µM of curcumin treatment, cell viability was significantly reduced to 70% and 84% at 48 h and 72 h, respectively. Cell viability less than 50% was observed at 25 µM (48 h and 72 h) and 50 µM (24 h) of MS13 treatment. In addition, MS13 and curcumin significantly showed greater inhibitory effect at 48 h and 72 h compared to 24 h at all concentrations. Overall, the results indicated that treatment with the MS13 demonstrated greater antiproliferative activity on NCI-H520 and NCI-H23 cells compared to curcumin in time- and dose-dependent manner. 

### 2.3. Morphological Observation of Apoptotic Cells by Acridine Orange

#### 2.3.1. Propidium Iodide (AO-PI) Double Staining

The apoptotic activity of MS13 on NCI-H520 and NCI-H23 cells was assessed through morphological observation by acridine orange (AO) and propidium iodide (PI) double staining method using fluorescent microscope. 

AO is an intercalating dye that can permeate viable and apoptotic cells, staining nuclear DNA in an intact cell membrane to generate green fluorescence. On the other hand, propidium iodide only penetrates dead cells with poor membrane integrity, staining all dead nucleated cells to generate red fluorescence. Therefore, every viable and apoptotic cell would exhibit different colors and characteristics upon AO/PI double staining [23,24]. Viable cells with intact membrane would exhibit uniform green nuclei, whereas early apoptotic cells would generate bright green nuclei with the additional characteristic of condensed or fragmented chromatin and membrane blebbing. Late apoptotic cells would exhibit yellow-orange to bright red fluorescence due to increased permeability of PI and condensed chromatin feature. As for necrotic cells, they would exhibit orange or red uniform nuclei since the cells would be fully permeable to AO and PI. 

**Figure 4:**
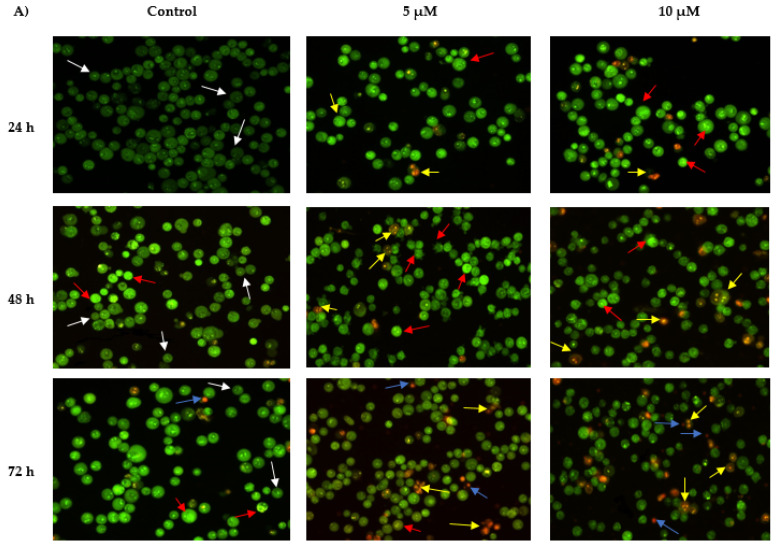

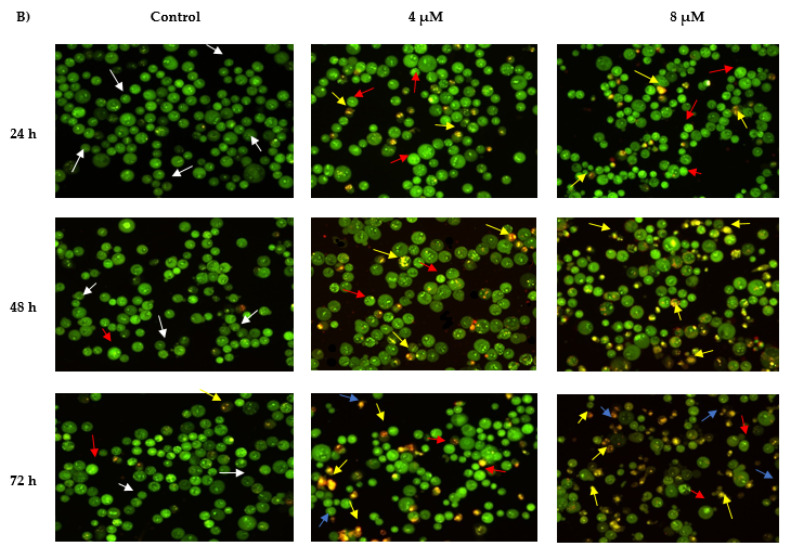
Detection by fluorescent microscope of acridine orange/propidium iodide double stained (**A**) NCI-H520 and (**B**) NCI-H23 cells treated with MS13 for 24, 48, and 72 h. Untreated viable cells are uniformly pale-green (white arrow). Early apoptotic cells showed characteristic loss of membrane integrity, membrane blebbing, and chromatin condensation, stained bright-green (red arrow). Late apoptotic cells stained yellow-orange to bright red, with a condensed or fragmented chromatin (yellow arrow). Necrotic cells showed orange or red uniform nuclei (blue arrow). Magnification is at 100×.

The morphological features of MS13-treated NCI-H520 and NCI-H23 cells morphological features are shown in Figure 4. Both treated cell lines showed a significant decrease in the appearance of viable cells and a notable increase in apoptotic cells as the treatment doses and time increase. In comparison with untreated cells (control), the morphological observation in the cell nuclei of treated cells showed various morphological alterations. In NCI-H520 cells (Figure 4A), treatment with 5 and 10 µM of MS13 showed an increased number of early apoptotic cells, which was indicated by bright green in appearance (red arrow). At 48 h, the treated cells for both doses demonstrated an increased number of early (red arrow) as well as late apoptotic cells (yellow arrow) compared to the treated cells at 24 h. However, at 72 h, the cells treated with 5 and 10 µM displayed a mixed population of late apoptotic and necrotic cells and an increased in number of yellow-orange to bright red stained cells compared to 48 h of treatment. Regarding NCI-H23 cells (Figure 4B), MS13 treatment at 4 and 8 µM of MS13 for 24 h displayed higher intensity of bright green fluorescent cells (red arrow) compared to the control, indicating a great number of early apoptotic cells present. However, at 48 h for both doses, a mixed population of cells bright green to yellow and small number of yellow-orange stained cells, indicating the presence of early and late apoptotic cells (red and yellow arrow) was observed. Upon 72 h of MS13 treatment at 8 µM, NCI-H23 cells displayed an increase of cells stained yellow–orange with condensed chromatin feature indicating the presence of late apoptotic cells as well as orange to red uniform nuclei indicating necrotic cells.

#### 2.3.2. Quantification of Apoptotic and Necrotic Cells

**Figure 5:**
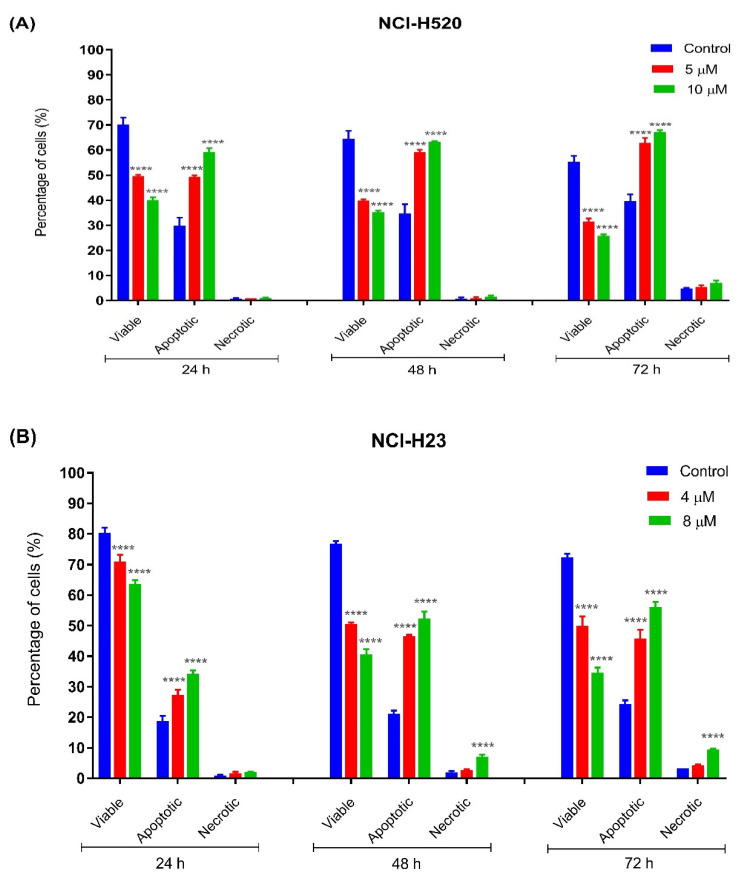
Percentage of cell population in (**A**) NCI-H520 and (**B**) NCI-H23 cells treated with MS13 for 24, 48, and 72 h. Treated and non-treated cells were double stained with AO/PI and a minimum of 200 cells were counted per sample and the percentage of cells from each population (viable, apoptotic, and necrotic) was calculated. Samples were run in triplicates and comparable results were obtained from three independent experiments. Comparison between data sets was performed using ANOVA. **** *p* < 0.0001 indicates statistically significant differences between the means of values obtained with treated vs. control.

The percentages of apoptotic and necrotic cells were also determined. For the purpose of analysis, early and late apoptosis were combined to indicate apoptotic activity. As observed in Figure 5, the percentage of viable cells decreased upon exposure to longer incubation times in both cell lines. Between the time points, there was an increase of apoptosis in all concentrations compared to the untreated samples (control). The results showed a comparatively increase percentage of cells undergoing apoptosis at 24, 48, as well as 72 h as treatment doses increased in both cell lines. 

As shown in Figure 5A, viable cells of untreated NCI-H520 cells with approximately more than 60% were noted at all time points (24, 48, and 72 h) compared to MS13-treated cells (5 and 10 µM). A significant decrease of viable cells in MS13-treated cells compared to untreated cells was noted at all time points by approximately 20–30% in a dose-dependent manner. The NCI-H520 cells treated with MS13 demonstrated a significant increase in apoptotic cells at 24, 48, and 72 h to approximately 50–60%, 60–65%, and 65–75%, respectively, in a dose-dependent manner. Higher apoptosis activity of NCI-H520 cells treated with MS13 was observed at incubation time of 48 and 72 h compared to 24 h. Although the apoptosis activity was relatively high at 48 and 72 h, the necrotic cells appeared to be low with less than 10% in the treated cells at these two points. Interestingly, no significant difference of necrotic cells was observed in NCI-H520 treated cells compared to untreated cells at both doses and time points.

As for NCI-H23 cells, more than approximately 70% of viable cells of untreated cells were noted at all time points (Figure 5B). The MS13 treatment with doses of 4 µM and 8 µM in NCI-H23 significantly reduced the viable cells to approximately 60–70% compared to untreated cells (80%) at 24 h in a dose-dependent manner. The percentage of MS13-treated viable cells at both doses further declined to approximately 45–55% and 35–50% at 48 and 72 h, respectively. A significant increase in apoptotic cells was noted in NCI-H23 cells following MS13 treatment at 24 h (25–35%), 48 and 72 h (45–55%). Higher apoptosis activity in NCI-H23 cells following MS13 treatment (4 µM and 8 µM) was observed at 48 and 72 h compared to 24 h; however, a significant increase in necrotic cells ranging from 8 to 15% was also noted at these time points for 8 µM MS13 treatment, whereas the percentage of necrotic cells at a dose of 4 µM remained low, with approximately less than 5% at all time points.

#### 2.3.3. Quantification of Relative Caspase-3 Activity

**Figure 6:**
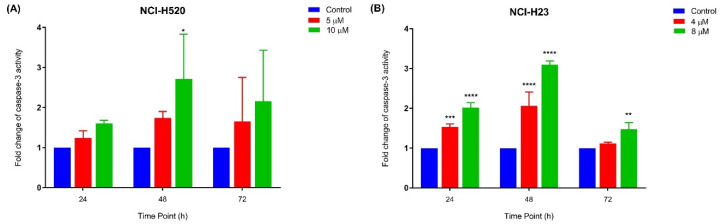
Relative caspase-3 activity in (**A**) NCI-H520 and (**B**) NCI-H23 cells treated with MS13 at different time points. Experiments were performed in triplicates and results compared between three independent experiments. Results are expressed as the ratio of means of caspase-3 activity of treated samples over untreated samples and comparison between data sets performed using ANOVA. Asterisks indicate statistically significant (* for *p <* 0.05, ** for *p* < 0.01, *** for *p <* 0.001 and **** for *p <* 0.0001) differences between data sets for each treatment dose.

The relative caspase-3 apoptotic activity was measured based on caspase-3 activity from the treated cells against the untreated cells (control). As depicted in Figure 6A, NCI-H520 cells only showed a significant increase in caspase-3 activity at 10 µM following 48 h of MS13 treatment. Meanwhile, there were no significant differences observed for the treated cells with 5 µM at all time points. As for NCI-H23 cells (Figure 6B), MS13 treatment at both doses following 24 and 48 h treatment demonstrated a significant increase in caspase-3 activity compared to control, whereas after 72 h of MS13 treatment, only treated cells at 10 µM showed a notable increase in caspase-3 activity. Taken together, these results indicate that MS13 induces apoptosis by caspase-3 activity in both cell lines in which NCI-H520 demonstrated an increase in caspase-3 activity at 10 µM following 48 h of treatment while NCI-H23 cells displayed a significant increase in caspase-3 activity at 24 and 48 h in a dose and time-dependent manner. 

#### 2.3.4. Quantification of Bcl-2 Protein Concentration

**Figure 7:**
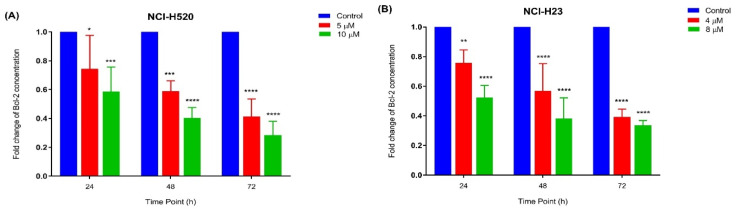
Fold change of Bcl-2 concentration in (**A**) NCI-H520 and (**B**) NCI-H23 cells treated with MS13 at different time points. Experiments were performed in triplicates and results compared between three independent experiments. Results are expressed as the ratio of means of Bcl-2 concentration of treated samples over untreated samples and comparison between datasets performed using ANOVA. Asterisks indicate statistically significant (* for *p <* 0.05, ** for *p* < 0.01 *** for *p <* 0.001 and **** for *p <* 0.0001) differences between data sets for each treatment dose.

The apoptotic activity of MS13 in NCI-H520 and NCI-H23 cells was further evaluated by assessing the Bcl-2 protein concentration. The treatment of NCI-H520 (Figure 7A) and NCI-H23 (Figure 7B) cells with MS13 (NCI-H520: 5 and 10 µM; NCI-H23: 4 and 8 µM) demonstrated a significant and progressive decrease of Bcl-2 concentration when treated for 24 to 72 h.

### 2.4. Analysis of Differentially Expressed Genes (DEGs) in MS13-Treated Lung Cancer Cells Associated with PI3K-AKT, Cell Cycle-Apoptosis, and MAPK Pathways

We further evaluated the gene expression profile of selected signaling pathways in MS13-treated NCI-H520 and NCI-H23 lung cancer cells using nCounter PanCancer Pathways panel. Based on the dosages previously established to induce apoptosis, NCI-H520 was treated with 10 μM of MS13 for 24 h, while NCI-H23 was treated with 4 μM of MS13 for 48 h. Only significant entities that demonstrated *p* < 0.05 and differentially expressed compared to the control by a fold change greater than or equal to 1.3 (FC ≥ 1.3) and fold change lower than or equal to −1.3 (FC ≤ −1.3) were considered for analysis. The gene expression data showed a total of 22 significant DEGs that mutually expressed in both NCI-H50 and NCI-H23 cells treated with MS13. However, a total of 77 and 47 significant (*p* < 0.05) DEGs were exclusively expressed in NCI-H520 and NCI-H23 cells treated with MS13, respectively. The total significant DEGs were further grouped to their respective signaling pathways as described by the nCounter PanCancer Pathways Code set. The 13 cancer-related pathways were NOTCH, APC/Wnt, Hedgehog, chromatin modification, transcriptional regulation, DNA damage control, TGF-Beta, MAPK, JAK-STAT, PI3K-AKT, driver genes, cell cycle-apoptosis, and Ras signaling pathways. Our results showed that the DEGs expressed in NCI-H520 and NCI-H23 cells treated with MS13 were highly associated with PI3K-AKT, cell cycle-apoptosis, and MAPK pathways. As shown in Table 2 and Table 3, these genes were associated with either a single pathway or multiple pathways. The findings showed that 12 DEGs were mutually expressed in both the cell lines, while 36 DEGs were exclusively expressed in NCI-H520 and 22 DEGs in NCI-H23 cells.

For the purpose of discussion, we selected the DEGs that demonstrated a fold change greater than or equal to 1.5 (FC ≥ 1.5) and fold change lower than or equal to −1.5 (FC ≤ −1.5). Among the mutually expressed DEGs, MCM7 associated with cell cycle-apoptosis, THBS1 with PI3K, MAPT with MAPK, and TGFB2 with cell cycle-apoptosis and MAPK were downregulated in both NCI-H520- and NCI-H23-treated cells. The DEG exclusively upregulated in NCI-H520 treated cells was ENDOG associated with cell cycle-apoptosis pathway. Meanwhile, exclusively downregulated DEGs included EPHA2, NGFR, MYB, ITGB4, COL4A5, KIT, and KITLG were associated with PI3K-AKT pathway, CACNA2D2, CACNA1E, DUSP4, MAP2K6, HSBP1, DUSP6, and RASGRP1 with MAPK pathway, and E2F1 with cell cycle-apoptosis pathway. Several exclusive DEGs expressed in MS13 treated-NCI-H520 cells were also associated with multiple pathways such as CDKN1A and PIK3R2 (cell cycle-apoptosis and PI3K-AKT pathways); GADD45G (cell cycle-apoptosis and MAPK pathways) and FGF9 and PRKCA (PI3K-AKT and MAPK pathways). In NCI-H23 cells treated with MS13, the exclusively downregulated DEGs were HGF, COL5A2, GNG4, MET, and ITGA3 associated with the PI3K-AKT pathway. Additionally, FGFR2, which is associated with multiple pathways (PI3K-AKT and MAPK pathways), was also exclusively downregulated in NCI-H23 cells following MS13 treatment.

## 3. Discussion

Our study showed that MS13 exhibited greater cytotoxicity and growth inhibitory effect in a dose-dependent manner at lower concentrations compared to the parent compound, curcumin, in both NCI-H520 and NCI-H23 cells. For cytotoxicity assay, the incubation time for MS13 treatment in NCI-H520 and NCI-H23 cells was selected as 72 h. Based on the doubling time of NCI-H520 and NCI-H23 cells, 72 h was the optimum time required for a maximal cytotoxic activity to occur compared to 24 and 48 h. MS13 was observed to have an increased inhibitory effect on NCI-H520 and NCI-H23 cells by approximately 5 times compared to curcumin based on the EC_50_ values. Previously, studies conducted by Citalingam et al. (2015), Paulraj et al. (2015), and Ismail et al. (2020), treating human prostate, cervical, and colon cancer cells with MS13, demonstrated greater inhibition effect with lower EC_50_ values compared to curcumin [19,20,21]. Similarly, previous studies also observed that colon, pancreatic, and gastric cancer cells treated with FLLL-11 [15,25] and GO-Y022 [26], which has identical structure as MS13 showed lower IC_50_ values compared to curcumin by several fold. In addition, other DAPs such as HO-3867 [16,27], EF24 [17,28], and GO-Y030 [29,30] demonstrated reduction in cell viability of human lung cancer cells compared to curcumin. It was also observed that NCI-H23 cells exhibited lower EC_50_ values compared to NCI-H520 cells following MS13 treatment. This finding suggests that NCI-H520 and NCI-H23 demonstrated differing sensitivities to MS13. This could be the result of being derived from different sources, as NCI-H520 is a squamous cell carcinoma while NCI-H23 is an adenocarcinoma. We believe that the different derivation of NCI-H520 and NCI-H23 may result in different biological, behavioral, and cell morphological characteristics which could contribute to the EC_50_ values noted upon MS13 treatment. Our results showed that SX values of MS13 treatment on NCI-H520 and NCI-H23 were above 100 indicating that MS13 is more selective towards lung cancer cells over the normal cells.

Antiproliferative assay was also performed over three time points (24, 48, and 72 h) to observe the dose and time-dependent effect of MS13 on NCI-H23 and NCI-H520 cells. The results revealed that MS13 caused a notable decline in cell viability of both cell lines as the time exposure and treatment dosage increased indicating inhibition of cell proliferation rate in a dose- and time-dependent manner. Vehicle-treated (DMSO) control cells demonstrated increased cell proliferation over time, while treatment with MS13 appears to have a significant inhibitory effect especially at later time points. The NCI-H520 cells displayed a substantial decrease of cell viability at 3.1 µM onwards for 48 and 72 h and at 6.3 µM onwards for 24 h. On the other hand, NCI-H23 cells showed a significant decrease of cell viability at 3.1 µM onwards for all time points. Thus, it can be suggested that MS13 treatment on NCI-H23 showed greater antiproliferative effect than NCI-H520 cells indicating different characteristics of cells respond differently to a treatment. Besides, curcumin was observed to exert an antiproliferative effect at a higher dosage of 25 µM onwards on both NCI-H520 and NCI-H23 lung cancer cells at all time points compared to MS13. Furthermore, it was observed that in all cases, MS13 treatment demonstrated greater growth inhibition compared to curcumin at 24, 48, and 72 h.

The enhanced cytotoxic and antiproliferative effects of MS13 against NCI-H520 and NCI-H23 cells compared to curcumin was believed due to the modification of the middle structure of curcumin which is the removal of β-diketone and substituents of 3′methoxy-4′-hydroxy on the phenyl rings [18,31]. It has been reported that the instability as well as decomposition of curcumin affected by alkaline pH and light exposure were caused by the active methylene moiety while the biological effects of curcumin were due to the role of hydroxy moiety at the aromatic rings [32]. Therefore, MS13 with mono-ketone derivative carrying α- and β-unsaturated ketone moiety is more stable and cause improved cytotoxicity and antiproliferative effects compared to curcumin.

Dysregulation in apoptotic pathways has been demonstrated to result in tumor cell formation as it creates a permissive environment for genetic pathway instability and accumulation of mutations [33]. Therefore, molecular pathway of apoptosis is regarded as the most potent strategy to counter the cancerous growth [34]. In the present study, morphological analysis and biochemical assays such as caspase 3 and bcl-2 were performed to determine the effect of MS13 on induction of apoptosis in NCI-H520 and NCI-H23 lung cancer cells. For apoptotic assays, MS13 at a dose of EC_50_ and 2× EC_50_ were treated on both cell lines at various time points (24, 48, and 72 h). Morphological analysis on apoptotic activity of MS13-treated NCI-H520 and NCI-H23 cells was performed using acridine orange (AO) and propidium iodide (PI) double staining method. Higher apoptotic activity of NCI-H520 and NCI-H23 cells treated with MS13 was observed at 48 and 72 h compared to 24 h for both dosages. Fluorescence microscopy analysis at 24 h revealed that NCI-H520 and NCI-H23 cells were stained bright green-yellow following treatment with both the doses, indicating induction of early apoptosis. Upon 48 h of MS13 treatment on both cells, there was a mixed population of cells with a larger proportion of early apoptotic cells compared to late suggesting that both early and late apoptotic events took place in both treated cells. Increase in late apoptotic cells and appearance of necrotic cells at 72 h of treatment resulted in higher percentage of cells stained bright orange-red. Nonetheless, the percentage of necrotic cells in both cells treated with MS13 at all doses remained low with less than 15% of total cell population. Taken together, these findings suggest that MS13 treatment at both doses induced apoptosis in NCI-H520 and NCI-H23 cells in dose and time-dependent manner with low percentage of necrotic cells.

Induction of apoptosis on lung cancer cells by MS13 treatment was further determined by caspase-3 activity and Bcl-2 protein concentration level. Caspase-3 is a cysteine protease that is activated early in a sequence of events associated with programmed cell death or apoptosis [35]. Activation of caspase-3 leads to cleavage of key substrate within the cell that is responsible to induce apoptosis eventually results in DNA degradation as well as morphological changes including cell blebbing, cell shrinkage, and chromatin condensation [36]. The present data showed that the increase in caspase-3 activity is dose-dependent with a significant increase being noted in NCI-H520 cells following MS13 treatment at a higher dose of 10 µM for 48 h. However, in NCI-H23 treated cells a significant increase was noted from 24 to 48 h but peaked at 48 h for both doses. Previous studies reported that MS13 induced apoptosis by caspase-3 activation in human cervical, prostate, and colon cancer cells [19,20,21]. Similarly, diarylpentanoids FLLL11 [15] and B19 [37] were shown to induce apoptosis in human colorectal cancer and ovarian cancer cells by increasing caspase-3 activity, respectively. Bcl-2 is a key protein in apoptosis induction by regulating mitochondrial membrane permeability and integrity, in addition to suppress cytochrome c release [38]. It has been reported that a decrease in bcl-2 levels leads to cell death by apoptosis while overexpression of bcl-2 protects cells from death [38,39]. NCI-H520 and NCI-H23 cells treated with MS13 for both doses at 24, 48, and 72 h exhibited a significant decrease in bcl-2 protein concentration compared to the untreated in dose and time-dependent manner. Previous studies reported similar findings that DAPs GO-Y030 [30] and DM1 [40] decreased bcl-2 protein concentration in colon cancer and melanoma cells, respectively. Therefore, our findings suggest that MS13 mediates the apoptotic activity in NCI-H520 and NCI-H23 lung cancer cells through the activation of caspase-3 and reduction of bcl-2 protein.

Gene expression analysis data showed that MS13 induced apoptosis in NCI-H520 and NCI-H23 cells by modulating genes associated with PI3K-AKT, cell cycle-apoptosis, and MAPK pathways. These signaling pathways play an essential role in the regulation of fundamental cellular functions such as transcription, translation, proliferation, growth, and survival. Accumulating evidences have shown that deregulation of these pathways is highly associated with various tumor development, including lung cancer. Previous studies demonstrated that the PI3K-Akt pathway is deregulated in lung cancer and has been associated with high-grade tumors, advanced disease, and poor prognosis [41]. Alteration of PI3K/Akt/mTOR pathway was found in 50–70% of NSCLC cases [42,43,44] and approximately 36% of SCLC cases [45]. Besides, the MAPK pathway also includes several proto-oncogenes and is deregulated in ~35% of lung cancer cases [46].

Our findings showed MCM7 (cell-cycle apoptosis), THBS1 (PI3K-AKT), MAPT (MAPK), and TGFB2 (cell-cycle apoptosis and MAPK) were mutually downregulated in NCI-H520 and NCI-H23 cells treated with MS13. The small number of mutual DEGs could be attributed to different biological and behavioral characteristic of NCI-H520 (squamous cell carcinoma) and NCI-H23 (adenocarcinoma) cells. MCM7 encodes minichromosome maintenance complex component 7 that is essential for the initiation and elongation of DNA replication [47]. MCM7 was associated with tumor development and progression in many cancers, including lung cancer [48,49,50,51]. Previous study reported that curcumin decreased the cell viability and altered the cell cycle of retinoblastoma cells by downregulating MCM7 [52]. MAPT codifies a microtubule-associated protein that promotes tubulin assembly and microtubule stabilization [53]. Overexpression of MAPT in colorectal [54], breast [55], and gastric [56] cancers was associated with tumor progression and poor prognosis. Previous study reported that higher expression of MAPT was detected in metastatic compared to primary prostate cancer patients [57]. TGFB2 encodes transforming growth factor-beta 2 protein that plays an important role in regulating cell proliferation, differentiation, motility, apoptosis, and immune regulation [58,59,60]. In renal cell carcinoma, knockdown of TGFB2 inhibited cell proliferation, migration, and invasion [61]. THBS1 encodes thrombospondin 1 (TSP1) protein which was shown to regulate cell growth and proliferation, cell motility, and cytoskeletal organization [62]. Previous studies have reported that TSP1 promoted angiogenesis, cell migration, and invasion in breast cancer cells [63,64]. The findings suggest that downregulation of MCM7, MAPT, TGFB2, and THBS1 by MS13 may inhibit cell proliferation and growth as well as metastasis and invasion in squamous cell carcinoma and adenocarcinoma NSCLC cells.

Our data demonstrated that ENDOG (cell cycle-apoptosis), CDKN1A (cell cycle-apoptosis and PI3K–AKT) and GADD45G (cell cycle-apoptosis and MAPK) genes were exclusively upregulated in MS13 treated NCI-H520 cells. The ENDOG gene encodes an apoptotic mitochondrial endonuclease G that translocates to the nucleus during apoptosis [65]. The ENDOG expression has been correlated with cancer cells sensitivity towards chemotherapeutic agents. ENDOG-positive breast cancer cells were more sensitive to chemotherapeutic agents compared to ENDOG-negative breast cancer cells [66]. Silencing ENDOG in ENDOG-positive prostate cancer cells decreased its sensitivity to chemotherapeutic agents [67]. This suggest that upregulation of ENDOG may increase sensitivity of NCI-H520 in response to MS13 treatment. Cyclin-Dependent Kinase Inhibitor 1A (CDKN1A), also known as p21 or WAF1, plays an important role in cell differentiation, proliferation, and apoptosis via regulating cell cycle [68,69]. Decreased expression of CDKN1A facilitates cell cycle progression from the G1 to S phase, thus promoting tumor cell proliferation [70,71]. Downregulation of CDKN1A was noted in several cancers including lung cancer [72,73,74] but upregulation of CDKN1A by various anticancer agents was reported to inhibit cell proliferation [75,76]. Curcumin inhibited cell proliferation in hepatocellular carcinoma cells through upregulation of CDKN1A [77]. Thus, we suggest that MS13 may inhibit cell proliferation in NCI-H520 cells through the upregulation of CDKN1A. GADD45G is a part of the growth arrest DNA damage-inducible gene (Gadd45g) family involved in DNA damage response and cell growth arrest [78]. Downregulation of GADD45G by aberrant promoter methylation has been noted in esophageal, colorectal, pancreatic, cervical, and lung cancer [79,80]. Decreased expression of GADD45G in esophageal cell carcinoma was correlated with tumor progression, metastasis and poor prognosis [81]. The study reported that GADD45G might act as a tumor suppressor gene and upregulation inhibits cell proliferation [81]. Therefore, MS13 may inhibit cell proliferation and metastasis in NCI-H520 cells through the upregulation of GADD45G.

Our results also showed that MS13 exclusively down-regulated EPHA2, ITGB4, COL4A5, KIT, KITLG, NGFR, and MYB, associated with PI3K-AKT pathway in NCI-H520 cells. The EPHA2, ITGB4, and COL4A5 genes have been associated with tumor progression by exhibiting cell invasion and metastasis. The EPHA2 gene encoding erythropoietin-producing hepatocellular A2 (EphA2) plays a significant role in cancer progression through neovascularization [82] while silencing of EPHA2 in human gastric cancer cells in vitro and in vivo decreased cell invasion, thus inhibiting cancer cell progression [83]. Curcumin was shown to inhibit tumor growth and angiogenesis in melanoma cells by downregulating EPHA2 [84]. ITGB4 encodes integrin α6β4 that regulates cell growth, motility, migration, invasion, and survival [85,86]. Aberrant expression of ITGB4 in lung [87], breast [88], and colorectal [89] cancers was associated with poor prognosis. Integrin α6β4 has been reported to stimulate cell invasion and metastasis in cancer cells through angiogenesis [90]. The COL4A5 gene encodes one of the Type IV collagen components that also plays a crucial role in angiogenesis, tissue remodeling, and cancer progression [91,92]. Col IV α5 deficiency delayed tumor progression in α5(IV)-deficient mouse model with lung tumor [93]. Therefore, the downregulation of EPHA2, ITGB4, and COL4A5 by MS13 in NCI-H520 may inhibit cell progression, metastasis, invasion, and angiogenesis. KIT encodes the tyrosine kinase receptor for kit ligand, also known as stem cell factor encoded by the KITLG gene. The interaction between KIT and KITLG plays an important role in normal cellular functions such as cell proliferation, differentiation, migration, and apoptosis as well as in tumorigenesis [94,95]. Krasagakis et al. noted that overexpression of both c-KIT and KITLG in Merkel cell carcinoma increased cell proliferation and decreased apoptosis [96]. This suggests that MS13 may inhibit cell proliferation and induce apoptosis by downregulating KIT and KITLG in NCI-H520 cells. NGFR codifies neurotrophin receptor that belongs to the tumor necrosis factor receptor superfamily [97]. It functions as either a promoter or suppressor of tumorigenesis. High expression of NGFR in melanoma [98,99], thyroid [100], and lung cancers [101] promoted cell proliferation and metastasis while reduced expression in liver [102], prostate [103], gastric [104], and bladder [105] cancers induced apoptosis. MYB encodes a transcription factor that regulates cell proliferation and cell differentiation [106]. Increased expression of MYB in colon cancer [107,108], breast cancer [109,110], and leukemias [111,112] was associated with cell proliferation, metastasis, and invasion. Therefore, MS13 may induce apoptosis while inhibit cell proliferation, metastasis and invasion in NCI-H520 cells by downregulating NGFR and MYB, respectively.

Our results showed that MAP2K6, HSBP1, RASGRP1, CACNA2D2, CACNA1E, DUSP4, and DUSP6 associated with MAPK pathway were downregulated in MS13 treated–NCI-H520 cells. The MAP2K6 gene encodes Mitogen-Activated Protein Kinase Kinase 6 (MAPKK6 also known as MKK6) acts as an upstream regulator of p38 MAPK signaling pathway [113,114]. Activation of p38 MAPK pathway has been shown to exert protumorigenic effect in prostate, esophageal, stomach, and colon cancers [115,116]. Overexpression of MKK6 was detected in 28% esophageal carcinoma biopsies and silencing the gene inhibited esophageal cancer cell proliferation [117]. Therefore, MS13 may inhibit cell proliferation in NCI-H520 cells by downregulating MAP2K6. HSBP1 encodes heat shock binding protein (HSBP1) which interacts with the heat shock factor (HSF1) and represses its transcriptional activity [118,119]. Shen et al. (2014) found that HSBP1 was overexpressed in oral squamous cell carcinoma (OSCC) tissue compared with its adjacent normal tissue. In this study, HSBP1-overexpressing cells showed resistance to radiotherapy, while HSBP1-repressed cells showed increased sensitivity to radiotherapy both in vivo and in vitro, suggesting that HSBP1 is critical for radioresistance of OSCC cells [120]. Therefore, the downregulation of HSBP1 by MS13 may contribute by increasing its sensitivity towards radiotherapy treatment. CACNA2D2 and CACNA1E encode calcium voltage-gated channel subunit alpha 2 delta 2 (α2δ2) and alpha 1e (α1e), respectively. The calcium ion channels play a crucial role in cell proliferation, differentiation, metastasis, and apoptosis [121]. Previous findings showed that CACNA2D2 promote tumorigenesis by stimulating cell proliferation and angiogenesis in prostate [122] and breast cancer [123] while CACNA1E was upregulated in lung squamous cell carcinoma [124] and wilms tumor [125]. Therefore, downregulation of CACNA2D2 and CACNA1E by MS13 may inhibit cell proliferation and metastasis and induce apoptosis in NCI-H520 cells. DUSP4 and DUSP6 genes encode Dual Specificity Protein Phosphatase and are members of the mitogen-activated protein kinase phosphatase (MKP) family that regulates the MAPK pathway [126]. Both DUSP4 and DUSP6 have been shown to induce cell proliferation, differentiation, and apoptosis [126,127,128]. The downregulation of DUSP4 in gastric cancer activated p53 signaling pathway, thus inducing apoptosis and cell cycle arrest at G2/M phase [129]. Additionally, overexpression of DUSP6 increased anchorage independent growth and invasion ability in immortal mouse melanocyte cell lines [130]. Our findings suggest that downregulation of both DUSP4 and DUSP6 by MS13 in NCI-H520 cells may induce apoptosis and inhibit cell growth and proliferation.

In our finding, the E2F1 gene associated with cell cycle-apoptosis pathway was downregulated by MS13 in NCI-H520 cells. E2F1 encodes one of the E2F protein family members that acts as a transcription factor and regulates G1/S phase transition [131]. Previous in vitro studies demonstrated that E2F1 has a tumor-promoting effect in lung [132,133], breast [134], and thyroid cancers [135]. Huang et al. (2007) revealed that E2F1 was positively correlated with thymidylate synthase (TS) and Survivin gene expressions in NSCLC. TS has been associated with tumor cell proliferation, chemotherapy resistance and poor prognosis but survivin gene with inhibition of apoptosis in NSCLC patients [136]. Therefore, the downregulation of E2F1 by MS13 may promote apoptosis and inhibit cell proliferation in NCI-H520 cells. Our findings also showed that PIK3R2 (cell cycle-apoptosis and PI3K pathways) and PRKCA (PI3K and MAPK pathways) associated with multiple pathways were downregulated in NCI-H520 cells treated with MS13. PIK3R2, a gene encoding p58β regulatory subunits that participated in most of the cancer-related and biological activity signaling pathways including PI3K, mTOR and cell cycle-apoptosis [137,138]. Elevated expression of p58β was noted in colon and breast cancers and positively associated with cell transformation, cell invasion, and tumor progression [139]. Hence, MS13 may inhibit cell transformation and invasion by downregulating PIK3R2 in NCI-H520 cells. PRKCA, a member of PKC family that plays a key role in regulating cell proliferation, survival and metastasis in cancers [140,141]. It has been indicated that PRKCA was highly elevated in NSCLC cells and associated with cell migration. Knockdown of PRKCA was shown to reduce the migration of NSCLC cells A549 compared to control cells [142]. Additionally, PRKCA expression was elevated in lung adenocarcinoma and positively associated with T classification, N classification, lymph node metastasis [143]. Thus, this finding suggests that the downregulation of PRKCA gene may inhibit cell metastasis and invasion in NCI-H520 cells.

Our results showed that MS13 downregulated HGF, MET, COL5A2, GNG4, and ITGA3 associated with PI3K-AKT pathway but FGFR2 was associated with multiple pathways (PI3K-AKT and MAPK) in NCI-H23 cells. HGF encodes a potent angiogenic cytokine that plays a vital role in angiogenesis by cooperating with vascular endothelial growth factor [144,145]. It has been indicated that HGF facilitates activation of MET signaling pathway [145] and our finding showed that MET was also downregulated in MS13-treated NCI-H23 cells. MET encodes c-Met, a tyrosine kinase receptor for HGF. Overexpression of c-Met was associated with cell proliferation, reduced apoptosis, angiogenesis, altered cytoskeletal function, and metastasis in some tumors including NSCLC [146,147,148]. Similarly, overexpression of HGF and/or its receptor c-Met have been reported in lung cancer cell lines and patients [149,150]. Curcumin was shown to inhibit epithelial–mesenchymal transition (EMT) and angiogenesis in HGF-induced lung cancer cells by regulating c-Met-dependent PI3K/Akt/mTOR signaling pathways [151]. Thus, it can be suggested that the downregulation of MET and HGF by MS13 may inhibit EMT and angiogenesis in NCI-H23 cells. COL5A2, a gene encoding a collagen type V α-2 chain has been associated with extracellular matrix organization, vascularization, EMTs process, invasion and metastasis in colorectal [152], breast [153], and bladder [154] cancers, and osteosarcoma [155]. Additionally, upregulation of COL5A2 was observed in lung adenocarcinoma compared to normal lung cells [156]. This suggests MS13 may inhibit cell invasion and metastasis of NCI-H23 cells through the downregulation of COL5A2. ITGA3 gene encodes an integrin alpha-3 subunit protein that serves as a cell surface adhesion molecule [157]. Increased expression of ITGA3 was noted in bladder, colorectal, pancreatic, prostate, nasopharyngeal carcinoma, and NSCLC cancers. Its elevated expression correlated with cancer metastasis through its interaction with extracellular matrix proteins [87,158,159,160,161,162]. Curcumin was reported to downregulate ITGA3 in lung cancer cells and cause inhibition of cell proliferation and invasion and induction of apoptosis [163]. Therefore, downregulation of ITGA3 by MS13 may inhibit cell proliferation and invasion and induce apoptosis in NCI-H23 cells. GNG4 encodes a member protein of the G protein family that involved in cancer development and growth [164,165,166]. Upregulation of GNG4 in lung [124], colon [167], and gastric [166] cancers was positively correlated with cell metastasis and poor prognosis. FGFR2 encodes two isoforms FGFR2b and FGFR2c and its activation is associated with angiogenesis and metastasis [168]. Li et al. (2018) demonstrated that FGFR2 expression was upregulated in cancer tissues compared to the adjacent normal tissues. The elevated expression of FGFR2 was correlated with lymph node metastasis and TNM stage, indicating its association with tumor invasion and metastasis [169]. This indicates that downregulation of GNG4 and FGFR2 by MS13 may inhibit cell invasion and metastasis in NCI-H23 cells.

## 4. Materials and Methods

### 4.1. Cell Culture and Maintenance

Two human lung cancer cell lines NCI-H520 (squamous cell carcinoma) and NCI-H23 (adenocarcinoma) as well as normal cell line MRC-9 (human lung fibroblast cells) were purchased from the American Type Culture Collection (ATCC, Manassas, VA, USA). Both NCI-H520 and NCI-H23 cell lines were grown in RPMI 1640 media while the MRC-9 cell line was grown in Eagle’s Minimum Essential Medium (EMEM, Gibco, Grand Island, NY, USA). All of the cell lines were maintained in respective media supplemented with 10% fetal bovine serum (FBS, Gibco) and 1% penicillin (100 U/mL)/streptomycin (100 µg/mL) (Gibco) in a humidified atmosphere with 5% CO_2_ at 37 °C. For sustaining the growth of the cells, the medium in the flask was changed at every two–three-day interval until 80–90% of growth confluency was achieved. Upon achieving 90% confluency, the cells were subcultured using accutase (Gibco) as the cell detachment solution. All of the cell culture procedures were performed in a biosafety cabinet and appropriate aseptic techniques were adhered strictly to prevent contamination.

### 4.2. Preparation of Curcumin Analogue (MS13) and Curcumin

Curcumin analog 1,5-bis(4-hydroxy-3-methoxyphenyl)-1,4-pentadien-3-one (MS13) was synthesized by coupling aromatic aldehyde with acetone and cyclohexone via base-catalyzed aldol condensation, in a 1:2 ratio of ketone to aldehyde [170]. Commercially available curcumin (Sigma-Aldrich, St. Louis, MA, USA) was used as a control against lung cancer as well as normal cell lines. Both MS13 and curcumin stock solution (50 mM) were prepared in dimethyl sulfoxide (DMSO, Sigma Aldrich, St. Louis, MA, USA).

### 4.3. Cell Viability and Antiproliferative Assays

Briefly, the cells were seeded in a flat-bottomed microtiter 96-well plates at concentration of 80,000 cells/mL with appropriate culture media in triplicates. Then, the cells were incubated in a 5% CO_2_ incubator at 37 ºC overnight to allow the cells to adhere to the bottom of the wells. After 24 h, the media was aspirated off and replaced with fresh media containing curcumin and MS13 at different concentrations ranging from 1.56 to 100 µM. Meanwhile, control wells contained untreated cells in appropriate media added with DMSO (0.2%). Both treated and untreated cells were incubated for 72 h for dose-dependent cytotoxicity assays and 24, 48, and 72 h for antiproliferative assay for both dose- and time-dependent. Upon completion, MTT [3-(4,5-dimethylthiazol-2-yl)-2,5-diphenyltetrazoliumbromide] assay was performed to determine the cell viability and antiproliferative activity. The media was aspirated and 100 µL media with 0.5 mg/mL MTT solution was added to each wells. The cells then were incubated for 4 h at 37 °C in a humidified 5% CO_2_ incubator. Following incubation, the excess MTT was discarded and 100 µL DMSO was added to each well to dissolve the formazan crystals. The absorbance of the blue formazan was read at 570/650 nm wavelength using a microplate spectrophotometer (BioTek™ EON™ Microplate Spectrophotometers, Fisher Scientific, Suwanee, GA, USA). The percentage of the cell viability was calculated as follows:

$$\mathrm{Cell}\;\mathrm{Viability}\;\%=\frac{\mathrm{Average}\;\mathrm{absorbance}\;\mathrm{of}\;\mathrm{treated}\;\mathrm{cell}}{\mathrm{Average}\;\mathrm{absorbance}\;\mathrm{of}\;\mathrm{untreatedcells}}\times 100$$

Based on the cell viability percentage, EC_50_ values were generated using the Graphpad prism version 7 software (Graphpad Software, La Jolla, CA, USA) with nonlinear regression curve fits of the data. The EC_50_ value indicates the concentration of MS13 required to reduce cell viability by 50% of the cell population. (R) The overall EC_50_ of each compound was determined based on the average EC_50_ values from three independent experiments. Selectivity index (SX) was determined based on the EC_50_ values obtained from cytotoxicity assays of MS13 against both normal and lung cancer cells. SX values was calculated based on the following equation as previously described (R):

$$\mathrm{Selectivity}\;\mathrm{Index}=\frac{{\mathrm{EC}}_{50}\left(\mathrm{Normal}\;\mathrm{cell}\;\mathrm{line}\right)}{{\mathrm{EC}}_{50}\left(\mathrm{Lung}\;\mathrm{cancer}\;\mathrm{cell}\;\mathrm{lines}\right)}\times 100$$

SX represents the reference to determine whether a selected compound possesses greater selectivity for achieving therapeutic effects in cancerous cells with minimal toxicity on normal cells (R.F). Selectivity index value above 100 indicates the tested compound possesses a high cytotoxic selectivity towards compared to normal cells [20].

### 4.4. Induction of Apoptosis by MS13

Apoptosis activity of MS13 against lung cancer cells NCI-H520 and NCI-H23 was assessed using morphological evaluation of apoptotic cells, caspase-3 activity, and bcl-2 cellular protein concentration. The NCI-H520 and NCI-H23 cells were treated with MS13 at approximate concentrations of its respective EC_50_ [NCI-H520, 5 µM; NCI-H23, 4 µM] and 2x EC_50_ [NCI-H520, 10 µM; NCI-H23, 8 µM] at 24, 48, and 72 h.

#### 4.4.1. Morphological Evaluation of Apoptotic Cells by Acridine Orange–Propidium Iodide (AO-PI) Double Staining

We used the double staining method using AO-PI to distinguish the morphology of viable, apoptotic, and necrotic cells. Briefly, both of NCI-H520 and NCI-H23 cells were seeded in T25 flasks (Nunc) and grown for 24 h. The test compound (MS13) of various concentrations (EC_50_ and 2× EC_50_) was added to each flask and incubated at 24, 48, and 72 h time intervals. Untreated cells containing DMSO alone were used as a negative control. Upon incubation, the cells were pelleted, washed twice in 1× PBS and resuspended in 150 µL 1× PBS. Prior to microscopic examination, each sample was mixed with 5 µL of acridine orange (50 µg/mL) and 5 µL of propidium iodide (50 µg/mL). The cell suspension and dye staining solution mixture was incubated at room temperature in the dark for 5 min. Twenty microliters of the cell suspension and dye mixture was placed on a glass microscopic slide covered with a cover slip. The stained cells were observed and photographed with a fluorescence microscope (BX41, Olympus, Melville, NY, USA) using dual filter set for FITC (green) and rhodomine (red). The experiment was conducted in triplicates. Upon completion, a minimum of 200 cells were counted per sample and the percentage of each cells (viable, apoptotic, and necrotic cells) from each population was calculated based on the following equation [24]:

$$\mathrm{Percentage}\;\mathrm{of}\;\mathrm{Cells}=\frac{\mathrm{No}\;\mathrm{of}\;\mathrm{viable}/\mathrm{apoptotic}/\mathrm{necrotic}\;\mathrm{cells}}{\mathrm{Total}\;\mathrm{targeted}\;\mathrm{cells}}\times 100$$

To assess the morphological criteria of viable, apoptotic, and necrotic cells, classification as follows was used [24]:

1. viable cells exhibit uniform green nuclei with intact structure
2. early apoptotic cells exhibit bright-green to yellow nuclei. In addition, characteristics of membrane integrity loss and chromatin condensation
3. late apoptotic cells exhibit yellow-orange to bright red nuclei as well as condensed or fragmented chromatin,
4. necrotic cells exhibit bright orange or red uniform nuclei.

#### 4.4.2. Caspase-3 Activity

Caspase-3 activity of MS13 on NCI-H520 cells was evaluated using the Caspase-3 Colorimetric Assay Kit (Raybiotech Inc., Peachtree Corners, GA, USA), following the protocol described by the manufacturer. The assay is based on spectrophotometric detection of *p*-nitroaniline (*p*NA), a colored molecule, after its cleavage from the labeled substrate DEVD-*p*NA by the activity of caspase-3 enzyme. Briefly, both cells were seeded and grown in T75 flasks (Nunc) until the growth reached 70–75% confluency. The cells were exposed to MS13 treatment at concentrations of EC_50_ and 2X EC_50_ for 24, 48, and 72 h. Following treatment, cells were washed with PBS and resuspended in 50 µL of chilled cell lysis buffer. The mixture of the cells and cell lysis buffer was incubated for 10 min on ice. After incubation, the cells were spun for 1 min at 10,000 g. Protein lysate concentrations were measured using bicinchoninic acid (BCA) protein assay kit (Thermo Fisher Scientific, USA) based on the manufacturer’s protocol. Upon protein quantification, 200 µg of protein lysate was diluted to 50 µL of cell lysis buffer for each sample. Next, 50 µL of 2× reaction buffer (containing 10 mM DTT) was added and followed by an additional of 5 µL of the 4 mM DEVD-*p*NA substrate to each assay. The mixture of each assay then was incubated at 37 °C for 2 h. The intensity of the color was measured at 405 nm in a microplate spectrophotometer (BioTek™ EON™ Microplate Spectrophotometers, Fisher Scientific, USA). Caspase-3 activity was expressed in fold change of absorbance from treated cells against absorbance from untreated cells as shown in following equation:

$$\mathrm{Fold}-\mathrm{change}=\frac{\mathrm{Absorbance}\;\mathrm{reading}\;\left(405\;\mathrm{nm}\right)\;\mathrm{of}\;\mathrm{treated}\;\mathrm{cells}}{\mathrm{Absorbance}\;\mathrm{reading}\;\left(405\;\mathrm{nm}\right)\;\mathrm{of}\;\mathrm{untreated}\;\mathrm{cells}}$$

#### 4.4.3. Bcl-2 Cellular Protein Concentration

Quantification of Bcl-2 cellular protein concentration was performed using the Human Bcl-2 ELISA kit (Invitrogen, Vienna, Austria) following the manufacturing instruction. Prior to the assay, NCI-H520 and NCI-H23 cells were cultured and treated with MS13 (EC_50_ and 2× EC_50_) for 24, 48, and 72 h. Then, the total protein was extracted from the treated cells and subjected to the Bcl-2 assay with each well of the supplied microtiter plate has been pre-coated with anti-human Bcl-2 antibody. Briefly, standards as well as samples were added to the wells and followed by Biotin conjugate. The plate then was incubated for 2 h at room temperature on microplate shaker. After incubation, the wells were washed to remove unbound material. Next, streptavidin-HRP was added to each well and incubated for an hour at room temperature. The wells were washed to remove unbound material and TMB substrate was added which reacted with the HRP enzyme resulting in color development. Last, stop solution was added to terminate color development reaction and the color intensity was measured at a wavelength of 450 nm by using a microplate spectrophotometer (BioTek™ EON™ Microplate Spectrophotometers, Fisher Scientific, USA). The data were presented in a fold-change of absorbance from treated cells against absorbance from untreated cells as shown in following equation:

$$\mathrm{Fold}-\mathrm{change}=\frac{\mathrm{Absorbance}\;\mathrm{reading}\;\left(450\;\mathrm{nm}\right)\;\mathrm{of}\;\mathrm{treated}\;\mathrm{cells}}{\mathrm{Absorbance}\;\mathrm{reading}\;\left(450\;\mathrm{nm}\right)\;\mathrm{of}\;\mathrm{untreated}\;\mathrm{cells}}$$

### 4.5. Gene Expression Analysis

#### 4.5.1. Total mRNA Extraction

Following treatment, the media was removed and washed with 1X PBS for three times. The cells were detached and washed with 1X PBS twice. Next, the cells were resuspended in 1 mL of 1X PBS and total mRNA was extracted from the harvested cells using RNeasy Mini Kit (Qiagen, Valencia, CA, USA) according to the manufacturer’s protocol. Concentration and purity of the extracted RNA was measured using the NanoDrop N60 spectrophotometer (Implen, Westlake Village, CA, USA). High-quality RNA samples with A260/280 ratios ranging from 1.7 to 2.3 and A260/230 ratios ranging from 1.8 to 2.3 were selected for gene expression analysis. The total mRNA content used for gene expression analysis were 75 ng. The samples were diluted to the concentration of 15 ng/ µL as 5 µL of sample was required for gene expression analysis.

#### 4.5.2. Nanostring nCounter Gene Expression Analysis

NanoString gene expression profiling was performed on RNA extracted from human lung cancer cells NCI-H520 and NCI-H2 using the nCounter PanCancer Pathways panel (NanoString Technologies, Seattle, WA, USA). The human PanCancer Pathways panel allows the evaluation of 770 genes (730 cancer related human genes, being 124 driver genes and 606 genes from 13 cancer-associated canonical pathways, and 40 as internal reference loci). This panel also contained 6 positive controls with concentrations ranging between 0.125–128 fM and 8 synthetic negative control sequences. Briefly, 75 ng of total RNA isolated from NCI-H520 and NCI-H23 cells were hybridized to specific capture and barcoded panel according to the manufacturer’s protocol. The hybridization reaction was incubated at 65 °C overnight before being immobilized on a cartridge.

#### 4.5.3. Sample Loading Protocol for nCounter SPRINT_TM_ Profiler

Briefly, the NanoString PanCancer cartridge from was equilibrated to room temperature for 15 min. Then, the hybridized samples were removed from the thermocycler and spun down. The RNAse-free water or hybridization buffer was added to sample for final volume of 30 µL, and 30 µL of each sample was loaded into the cartridge. A transparent cover sheet was then sealed over the sample loading ports and the protective green seal was removed from the reagent ports. Finally, the cartridge was placed into the cartridge drawer. Before running the instrument, cartridge was ensured to align in the proper orientation and fully seated in the cartridge tray.

#### 4.5.4. Data Collection and Data Analysis

A nCounter Digital Analyzer was used to count the fluorescent barcoded probes to quantify each target RNA molecule. The barcoded images captured by the automated fluorescent microscope were preprocessed for quality control metrics based on field of view (FOV) registration and binding density. Processing and normalization of raw NanoString gene expression data were conducted using the nSolver Advanced Analysis Software (NanoString Technologies^TM^). Significant entities that demonstrated a *p* < 0.05 and differentially expressed genes (DEGs) with fold change greater than or equal to 1.3 (FC ≥ 1.3) and lower than or equal to −1.3 (FC ≤ −1.3) were considered as significant DEGs. Comparisons between the control and the treatment groups for both NCI-H520 and NCI-H23 cells were performed.

## 5. Conclusions

In summary, MS13 demonstrated greater dose-dependent cytotoxicity and dose- and time-dependent antiproliferative activity compared to curcumin in NCI-H520 and NCI-H23 cells. The morphological observation, increase in caspase-3 activity, as well as progressive decrease in Bcl-2 expression indicated induction of apoptosis by MS13. The gene expression analysis revealed that the DEGs modulated by MS13 in NCI-H520 and NCI-H23 cells were highly associated with PI3K, cell cycle-apoptosis, and MAPK signaling pathways. The findings suggest that MS13 may induce antiproliferation and apoptosis activity in squamous cell carcinoma and adenocarcinoma of NSCLC cells by modulating DEGs associated with PI3K-AKT, cell cycle-apoptosis, and MAPK pathways. Therefore, the present study could provide an insight into the anticancer activity of MS13 and merits further investigation as a potential anticancer agent for NSCLC cancer therapy.

## Figures and Tables

**Table 1:** EC_50_ values of curcumin and MS13 on NCI-H520, NCI-H23, and MRC9 cells and their selective indices.

Compounds	EC_50_ Values (µM)			Selective Index (SX)	
	NCI-H520	NCI-H23	MRC9	NCI-H520	NCI-H23
MS13	4.7 ± 0.1	3.7 ± 0.4	8.6 ± 0.6	181.4	232.4
Curcumin	25.19 ± 1.7	18.5 ± 0.7	27.4 ± 1.7	108.8	148.2

Results are shown as mean ± standard deviation (S.D) from three independent experiments.

**Table 2:** Upregulated DEGs in NCI-H520 and NCI-H23 cells in response to MS13 treatment.

Genes/Cell Line	Gene Description	Accession No.	Single/Multiple Pathways	FC	*p*-Value
**NCI-H520**					
PTEN	Phosphatase and Tensin Homolog	NM_000314.3:1675	PI3K	1.3	0.0233
DDIT4	DNA Damage Inducible Transcript 4	NM_019058.2:85	PI3K	1.4	0.0223
ENDOG	Endonuclease G	NM_004435.2:694	Cell-cycle Apoptosis	1.5	0.00367
CASP8	Caspase 8	NM_001228.4:301	Cell-cycle Apoptosis	1.4	0.0341
DDIT3	DNA Damage Inducible Transcript 3	NM_004083.4:40	MAPK	1.3	0.00325
CDKN1A	Cyclin Dependent Kinase Inhibitor 1A	NM_000389.2:1975	Cell-cycle Apoptosis and PI3K	2.1	0.0006
* CCND3	Cyclin D3	NM_001760.2:1215	Cell-cycle Apoptosis and PI3K	1.4	0.015
GADD45G	Growth Arrest and DNA Damage Inducible Gamma	NM_006705.3:250	Cell-cycle Apoptosis and MAPK	2.1	0.000258
**NCI-H23**					
ITGA2	Integrin Subunit Alpha 2	NM_002203.2:475	PI3K	1.4	0.00405
THEM4	Thioesterase Superfamily Member 4	NM_053055.4:764	PI3K	1.3	0.00716
TNFRSF10A	TNF Receptor Superfamily Member 10a	NM_003840.3:2380	Cell-cycle Apoptosis	1.4	0.00918
IRAK2	Interleukin 1 Receptor Associated Kinase 2	NM_001570.3:1285	Cell-cycle Apoptosis	1.4	0.00186
DUSP2	Dual Specificity Phosphatase 2	NM_004418.3:1235	MAPK	1.3	0.0344
* CCND3	Cyclin D3	NM_001760.2:1215	Cell-cycle Apoptosis and PI3K	1.3	0.00733

* Indicates mutually expressed DEGs in both NCI-H520 and NCI-H23 cells treated with MS13.

**Table 3:** Downregulated DEGs in NCI-H520 and NCI-H23 cells in response to MS13 treatment.

Genes/Cell Line	Gene Description	Accession No.	Single/Multiple Pathways	FC	*p*-Value
**NCI-H520**					
EPHA2	EPH Receptor A2	NM_004431.2:1525	PI3K	−2.0	0.00397
NGFR	Nerve Growth Factor Receptor	NM_002507.1:2705	PI3K	−2.0	0.00691
MYB	MYB Proto-Oncogene, Transcription Factor	NM_005375.2:3145	PI3K	−1.9	0.00042
ITGB4	Integrin Subunit Beta 4	NM_001005731.1:4151	PI3K	−1.7	0.00064
COL4A5	Collagen Type IV Alpha 5 Chain	NM_033381.1:5360	PI3K	−1.7	0.00275
* THBS1	Thrombospondin 1	NM_003246.2:3465	PI3K	−1.7	0.00044
KIT	KIT Proto-Oncogene, Receptor Tyrosine Kinase	NM_000222.1:5	PI3K	−1.6	0.0418
KITLG	KIT Ligand	NM_003994.4:1155	PI3K	−1.5	0.00058
PDGFD	Platelet Derived Growth Factor D	NM_025208.4:1120	PI3K	−1.4	0.0496
FN1	Fibronectin 1	NM_212482.1:1776	PI3K	−1.3	0.00927
ITGA2	Integrin Subunit Alpha 2	NM_002203.2:475	PI3K	−1.3	0.0199
* MCM-7	Minichromosome Maintenance Complex Component 7	NM_182776.1:1325	Cell-cycle Apoptosis	−4.3	1.62 × 10^−5^
E2F1	E2F Transcription Factor 1	NM_005225.1:935	Cell-cycle Apoptosis	−1.5	0.00051
MAP2K1	Mitogen-Activated Protein Kinase Kinase 1	NM_002755.2:970	Cell-cycle Apoptosis	−1.4	0.00012
CDKN2C	Cyclin Dependent Kinase Inhibitor 2C	NM_001262.2:1295	Cell-cycle Apoptosis	−1.4	0.013
PRKAR1B	Protein Kinase CAMP-Dependent Type I Regulatory Subunit Beta	NM_001164759.1:1112	Cell-cycle Apoptosis	−1.4	0.00524
CDKN2D	Cyclin-Dependent Kinase Inhibitor 2D	NM_001800.3:870	Cell-cycle Apoptosis	−1.4	0.0333
CASP7	Caspase 7	NM_001227.3:915	Cell-cycle Apoptosis	−1.3	0.0198
PKMYT1	Protein Kinase, Membrane-Associated Tyrosine/Threonine 1	NM_004203.3:780	Cell-cycle Apoptosis	−1.3	0.00813
HDAC10	Histone Deacetylase 1	NM_032019.5:932	Cell-cycle Apoptosis	−1.3	0.00159
MYD88	Innate Immune Signal Transduction Adaptor	NM_002468.3:2145	Cell-cycle Apoptosis	−1.3	0.00872
* SKP2	S-Phase Kinase-Associated Protein 2	NM_005983.2:615	Cell-cycle Apoptosis	−1.3	0.00401
CACNA2D2	Calcium Voltage-Gated Channel Auxiliary Subunit Alpha2delta 2	NM_001005505.1:2045	MAPK	−2.1	0.0113
CACNA1E	Calcium Voltage-Gated Channel Subunit Alpha1 E	NM_000721.2:9325	MAPK	−2.1	0.00435
DUSP4	Dual Specificity Phosphatase 4	NM_057158.2:3115	MAPK	−1.6	0.0319
MAP2K6	Mitogen-Activated Protein Kinase Kinase 6	NM_002758.3:555	MAPK	−1.5	0.00795
* MAPT	Microtubule Associated Protein Tau	NM_016834.3:1205	MAPK	−1.5	0.0469
THSBP1	Heat Shock Factor Binding Protein 1	NM_003246.2:3465	MAPK	−1.5	0.0123
DUSP6	Dual Specificity Phosphatase 6	NM_001946.2:1535	MAPK	−3.3	6.93 × 10^−5^
RASGRP1	RAS Guanyl Releasing Protein 1	NM_005739.3:365	MAPK	−1.7	0.0297
PIK3R2	Phosphoinositide-3-Kinase Regulatory Subunit 2	NM_005027.2:3100	Cell cycle-apoptosis and PI3K	−1.5	0.00943
BAD	BCL2 Associated Agonist of Cell Death	NM_004322.3:652	Cell cycle-apoptosis and PI3K	−1.4	0.00012
CDK6	Cyclin-Dependent Kinase 6	NM_001259.5:15	Cell cycle-apoptosis and PI3K	−1.4	0.00398
PIK3R3	Phosphoinositide-3-Kinase Regulatory Subunit 3	NM_003629.3:1800	Cell cycle-apoptosis and PI3K	−1.3	0.00023
* PIK3CB	Phosphatidylinositol-4,5-Bisphosphate 3-Kinase Catalytic Subunit Beta	NM_006219.1:2945	Cell cycle-apoptosis and PI3K	−1.3	0.00023
FGF9	Fibroblast Growth Factor 9	NM_002010.2:1565	PI3K and MAPK	−1.8	0.00742
PRKCA	Protein Kinase C Alpha	NM_002737.2:5560	PI3K and MAPK	−1.6	0.0235
MAP2K1	Mitogen-Activated Protein Kinase Kinase 1	NM_002755.2:970	PI3K and MAPK	−1.4	0.000117
MAP2K2	Mitogen-Activated Protein Kinase Kinase 2	NM_030662.2:1325	PI3K and MAPK	−1.4	0.00258
* MAPK3	Mitogen-Activated Protein Kinase 3	NM_001040056.1:580	PI3K and MAPK	−1.3	0.016
* TGFB2	Transforming Growth Factor Beta 2	NM_003238.2:1125	Cell cycle-apoptosis and MAPK	−1.5	0.00123
PRKACA	Protein Kinase CAMP-Activated Catalytic Subunit Alpha	NM_002730.3:400	Cell cycle-apoptosis, PI3K and MAPK	−1.3	4.50 × 10^−5^
AKT1	AKT Serine/Threonine Kinase 1	NM_005163.2:1772	Cell cycle-apoptosis, PI3K and MAPK	−1.3	0.0007
**NCI-H23**					
HGF	Hepatocyte Growth Factor	NM_000601.4:550	PI3K	−1.8	0.0285
COL5A2	Collagen Type V Alpha 2 Chain	NM_000393.3:4075	PI3K	−1.7	0.00464
GNG4	G Protein Subunit Gamma 4	NM_004485.2:215	PI3K	−1.6	0.028
MET	MET Proto-Oncogene, Receptor Tyrosine Kinase	NM_000245.2:405	PI3K	−1.5	0.00293
ITGA3	Integrin Subunit Alpha 3	NM_005501.2:1138	PI3K	−1.5	0.0131
* THBS1	Thrombospondin 1	NM_003246.2:3465	PI3K	−1.4	0.00578
IRS1	Insulin Receptor Substrate 1	NM_005544.2:6224	PI3K	−1.4	0.00412
EFNA3	Ephrin A3	NM_004952.4:1672	PI3K	−1.4	0.0205
EIF4EBP1	Eukaryotic Translation Initiation Factor 4E Binding Protein 1	NM_001429.2:715	PI3K	−1.3	0.0184
* MCM-7	Minichromosome Maintenance Complex Component 7	NM_182776.1:1325	Cell-cycle Apoptosis	−1.7	0.00101
CCNA1	Cyclin A1	NM_003914.3:1605	Cell-cycle Apoptosis	−1.4	0.0264
SMAD2	SMAD Family Member 2	NM_001003652.1:4500	Cell-cycle Apoptosis	−1.4	0.0199
CHEK2	Checkpoint Kinase 2	NM_007194.3:140	Cell-cycle Apoptosis	−1.3	0.0398
MAD2L2	Mitotic Arrest Deficient 2 Like 2	NM_001127325.1:290	Cell-cycle Apoptosis	−1.3	0.0128
* SKP2	S-Phase Kinase Associated Protein 2	NM_005983.2:615	Cell-cycle Apoptosis	−1.3	0.00026
CDC7	Cell Division Cycle 7	NM_003503.2:805	Cell-cycle Apoptosis	−1.3	0.0302
RAD21	RAD21 Cohesin Complex Component	NM_006265.2:1080	Cell-cycle Apoptosis	−1.3	0.0289
* MAPT	Microtubule Associated Protein Tau	NM_016834.3:1205	MAPK	−1.5	0.0469
RAC3	Rac Family Small GTPase 3	NM_005052.2:702	MAPK	−1.4	0.0439
FLNA	Filamin A	NM_001456.3:7335	MAPK	−1.3	0.00262
* PIK3CB	Phosphatidylinositol-4,5-Bisphosphate 3-Kinase Catalytic Subunit Beta	NM_006219.1:2945	Cell cycle-apoptosis and PI3K	−1.3	0.0272
FAS	Fas Cell Surface Death Receptor	NM_152876.1:1740	Cell cycle-apoptosis and MAPK	−1.4	0.00328
PPP3CA	Protein Phosphatase 3 Catalytic Subunit Alpha	NM_000944.4:3920	Cell cycle-apoptosis and MAPK	−1.3	0.0223
* TGFB2	Transforming Growth Factor Beta 2	NM_003238.2:1125	Cell cycle-apoptosis and MAPK	−1.6	0.0104
FGFR2	Fibroblast Growth Factor Receptor 2	NM_000141.4:647	PI3K and MAPK	−1.6	0.0265
FGF2	Fibroblast Growth Factor 2	NM_002006.4:620	PI3K and MAPK	−1.4	0.00307
FGFR4	Fibroblast Growth Factor Receptor 4	NM_002011.3:1585	PI3K and MAPK	−1.3	0.027
* MAPK3	Mitogen-Activated Protein Kinase 3	NM_001040056.1:580	PI3K and MAPK	−1.4	0.0425
TP53	Tumor Protein P53	NM_000546.2:1330	Cell cycle-apoptosis, PI3K and MAPK	−1.3	0.00783

* Indicates mutually expressed DEGs in both NCI-H520 and NCI-H23 cells treated with MS13.

## Data Availability

The data presented in this study are available within the article.

## References

[1] Sung H., Ferlay J., Siegel R.L., Laversanne M., Soerjomataram I., Jemal A., Bray F. (2021). Global cancer statistics 2020: GLOBOCAN estimates of incidence and mortality worldwide for 36 cancers in 185 countries. CA Cancer J. Clin..

[2] Travis W.D. (2011). Pathology of lung cancer. Clin. Chest Med..

[3] Minna J.D., Roth J.A., Gazdar A.F. (2002). Focus on lung cancer. Cancer Cell.

[4] Miller K.D., Siegel R.L., Lin C.C., Mariotto A.B., Kramer J.L., Rowland J.H., Stein K.D., Alteri R., Jemal A. (2016). Cancer treatment and survivorship statistics, 2016. CA Cancer J. Clin..

[5] Arriagada R., Dunant A., Pignon J.-P., Bergman B., Chabowski M., Grunenwald D., Kozlowski M., Le Péchoux C., Pirker R., Pinel M.-I.S. (2009). Long-Term Results of the International Adjuvant Lung Cancer Trial Evaluating Adjuvant Cisplatin-Based Chemotherapy in Resected Lung Cancer. J. Clin. Oncol..

[6] Altaf M.M., Ahmad Khan M.S., Ahmad I., Ahmad Khan M.S., Ahmad I., Chattopadhyay D. (2019). Chapter 2—Diversity of Bioactive Compounds and Their Therapeutic Potential. New Look to Phytomedicine.

[7] Panahi Y., Hosseini M.S., Khalili N., Naimi E., Simental-Mendía L.E., Majeed M., Sahebkar A. (2016). Effects of curcumin on serum cytokine concentrations in subjects with metabolic syndrome: A post-hoc analysis of a randomized controlled trial. Biomed. Pharmacother..

[8] Sahebkar A., Serban M.-C., Ursoniu S., Banach M. (2015). Effect of curcuminoids on oxidative stress: A systematic review and meta-analysis of randomized controlled trials. J. Funct. Foods.

[9] Moghadamtousi S.Z., Kadir H.A., Hassandarvish P., Tajik H., Abubakar S., Zandi K. (2014). A Review on Antibacterial, Antiviral, and Antifungal Activity of Curcumin. BioMed Res. Int..

[10] Vallianou N.G., Evangelopoulos A., Schizas N., Kazazis C. (2015). Potential anticancer properties and mechanisms of action of curcumin. Anticancer. Res..

[11] Mehta H.J., Patel V., Sadikot R.T. (2014). Curcumin and lung cancer—A review. Target. Oncol..

[12] Wan Mohd Tajuddin W.N.B., Lajis N.H., Abas F., Othman I., Naidu R. (2019). Mechanistic understanding of curcumin’s therapeutic effects in lung cancer. Nutrients.

[13] Anand P., Kunnumakkara A.B., Newman R.A., Aggarwal B.B. (2007). Bioavailability of curcumin: Problems and promises. Mol. Pharm..

[14] Paulraj F., Abas F., Lajis N.H., Othman I., Naidu R. (2019). Molecular Pathways Modulated by Curcumin Analogue, Diarylpentanoids in Cancer. Biomolecules.

[15] Cen L., Hutzen B., Ball S., DeAngelis S., Chen C.-L., Fuchs J.R., Li C., Li P.-K., Lin J. (2009). New structural analogues of curcumin exhibit potent growth suppressive activity in human colorectal carcinoma cells. BMC Cancer.

[16] Selvendiran K., Ahmed S., Dayton A., Kuppusamy M.L., Tazi M., Bratasz A., Tong L., Rivera B.K., Kálai T., Hideg K. (2010). Safe and targeted anticancer efficacy of a novel class of antioxidant-conjugated difluorodiarylidenyl piperidones: Differential cytotoxicity in healthy and cancer cells. Free Radic. Biol. Med..

[17] Tan X., Sidell N., Mancini A., Huang R.-P., Shenming W., Horowitz I.R., Liotta D.C., Taylor R.N., Wieser F. (2010). Multiple Anticancer Activities of EF24, a Novel Curcumin Analog, on Human Ovarian Carcinoma Cells. Reprod. Sci..

[18] Liang G., Shao L., Wang Y., Zhao C., Chu Y., Xiao J., Zhao Y., Li X., Yang S. (2009). Exploration and synthesis of curcumin analogues with improved structural stability both in vitro and in vivo as cytotoxic agents. Bioorganic Med. Chem..

[19] Citalingam K., Abas F., Lajis N.H., Othman I., Naidu R. (2015). Anti-proliferative effect and induction of apoptosis in androgen-independent human prostate cancer cells by 1, 5-bis (2-hydroxyphenyl)-1, 4-pentadiene-3-one. Molecules.

[20] Paulraj F., Abas F., Lajis N.H., Othman I., Hassan S.S., Naidu R. (2015). The curcumin analogue 1, 5-bis (2-hydroxyphenyl)-1, 4-pentadiene-3-one induces apoptosis and downregulates E6 and E7 oncogene expression in HPV16 and HPV18-infected cervical cancer cells. Molecules.

[21] Ismail N.I., Othman I., Abas F., Lajis N.H., Naidu R. (2020). The Curcumin Analogue, MS13 (1, 5-Bis (4-hydroxy-3-methoxyphenyl)-1, 4-pentadiene-3-one), Inhibits Cell Proliferation and Induces Apoptosis in Primary and Metastatic Human Colon Cancer Cells. Molecules.

[22] Lee Y.Q., Rajadurai P., Abas F., Othman I., Naidu R. (2021). Proteomic Analysis on Anti-Proliferative and Apoptosis Effects of Curcumin Analog, 1, 5-bis (4-Hydroxy-3-Methyoxyphenyl)-1, 4-Pentadiene-3-One-Treated Human Glioblastoma and Neuroblastoma Cells. Front. Mol. Biosci..

[23] Ng K.-B., Bustamam A., Sukari M.A., Abdelwahab S.I., Mohan S., Buckle M.J.C., Kamalidehghan B., Nadzri N.M., Anasamy T., Hadi A.H.A. (2013). Induction of selective cytotoxicity and apoptosis in human T4-lymphoblastoid cell line (CEMss) by boesenbergin a isolated from boesenbergia rotunda rhizomes involves mitochondrial pathway, activation of caspase 3 and G2/M phase cell cycle arrest. BMC Complement. Altern. Med..

[24] Zimmer S., Kahl P., Buhl T.M., Steiner S., Wardelmann E., Merkelbach-Bruse S., Buettner R., Heukamp L.C. (2009). Epidermal growth factor receptor mutations in non-small cell lung cancer influence downstream Akt, MAPK and Stat3 signaling. J. Cancer Res. Clin. Oncol..

[25] Friedman L., Lin L., Ball S., Bekaii-Saab T., Fuchs J., Li P.-K., Li C., Lin J. (2009). Curcumin analogues exhibit enhanced growth suppressive activity in human pancreatic cancer cells. Anti-Cancer Drugs.

[26] Yoshida T., Maruyama T., Miura M., Inoue M., Fukuda K., Shimazu K., Taguchi D., Kanda H., Oshima M., Iwabuchi Y. (2018). Dietary intake of pyrolyzed deketene curcumin inhibits gastric carcinogenesis. J. Funct. Foods.

[27] Selvendiran K., Tong L., Bratasz A., Kuppusamy M.L., Ahmed S., Ravi Y., Trigg N.J., Rivera B.K., Kálai T., Hideg K. (2010). Anticancer efficacy of a difluorodiarylidenyl piperidone (HO-3867) in human ovarian cancer cells and tumor xenografts. Mol. Cancer Ther..

[28] Subramaniam D., May R., Sureban S.M., Lee K.B., George R., Kuppusamy P., Ramanujam R.P., Hideg K., Dieckgraefe B.K., Houchen C.W. (2008). Diphenyl difluoroketone: A curcumin derivative with potent in vivo anticancer activity. Cancer Res..

[29] Hutzen B., Friedman L., Sobo M., Lin L., Cen L., De Angelis S., Yamakoshi H., Shibata H., Iwabuchi Y., Lin J. (2009). Curcumin analogue GO-Y030 inhibits STAT3 activity and cell growth in breast and pancreatic carcinomas. Int. J. Oncol..

[30] Lin L., Liu Y., Li H., Li P., Fuchs J., Shibata H., Iwabuchi Y., Lin J. (2011). Targeting colon cancer stem cells using a new curcumin analogue, GO-Y030. Br. J. Cancer.

[31] Lin L., Shi Q., Nyarko A.K., Bastow K.F., Wu C.-C., Su C.-Y., Shih C.C.-Y., Lee K.-H. (2006). Antitumor agents. 250. Design and synthesis of new curcumin analogues as potential anti-prostate cancer agents. J. Med. Chem..

[32] Van der Goot H. (1997). The chemistry and qualitative structure-activity relationships of curcumin. Recent Developments in Curcumin Pharmacochemistry.

[33] Danial N.N., Korsmeyer S.J. (2004). Cell death: Critical control points. Cell.

[34] Wong R.S. (2011). Apoptosis in cancer: From pathogenesis to treatment. J. Exp. Clin. Cancer Res..

[35] Porter A.G., Jänicke R.U. (1999). Emerging roles of caspase-3 in apoptosis. Cell Death Differ..

[36] Jänicke R.U., Sprengart M.L., Wati M.R., Porter A.G. (1998). Caspase-3 is required for DNA fragmentation and morphological changes associated with apoptosis. J. Biol. Chem..

[37] Qu W., Xiao J., Zhang H., Chen Q., Wang Z., Shi H., Gong L., Chen J., Liu Y., Cao R. (2013). B19, a novel monocarbonyl analogue of curcumin, induces human ovarian cancer cell apoptosis via activation of endoplasmic reticulum stress and the autophagy signaling pathway. Int. J. Biol. Sci..

[38] Cory S., Adams J.M. (2002). The Bcl2 family: Regulators of the cellular life-or-death switch. Nat. Rev. Cancer.

[39] Kim M.E., Ha T.K., Yoon J.H., Lee J.S. (2014). Myricetin induces cell death of human colon cancer cells via BAX/BCL2-dependent pathway. Anticancer. Res..

[40] Faião-Flores F., Suarez J.A.Q., Soto-Cerrato V., Espona-Fiedler M., Pérez-Tomás R., Maria D.A. (2013). Bcl-2 family proteins and cytoskeleton changes involved in DM-1 cytotoxic effect on melanoma cells. Tumor Biol..

[41] Sarris E.G., Saif M.W., Syrigos K.N. (2012). The Biological Role of PI3K Pathway in Lung Cancer. Pharmaceuticals.

[42] Papadimitrakopoulou V. (2012). Development of PI3K/AKT/mTOR pathway inhibitors and their application in personalized therapy for non–small-cell lung cancer. J. Thorac. Oncol..

[43] Tsurutani J., Fukuoka J., Tsurutani H., Shih J.H., Hewitt S.M., Travis W.D., Jen J., Dennis P.A. (2006). Evaluation of two phosphorylation sites improves the prognostic significance of Akt activation in non–small-cell lung cancer tumors. J. Clin. Oncol..

[44] Tang J.-M., He Q.-Y., Guo R.-X., Chang X.-J. (2006). Phosphorylated Akt overexpression and loss of PTEN expression in non-small cell lung cancer confers poor prognosis. Lung Cancer.

[45] Umemura S., Mimaki S., Makinoshima H., Tada S., Ishii G., Ohmatsu H., Niho S., Yoh K., Matsumoto S., Takahashi A. (2014). Therapeutic priority of the PI3K/AKT/mTOR pathway in small cell lung cancers as revealed by a comprehensive genomic analysis. J. Thorac. Oncol..

[46] Sebolt-Leopold J.S., Herrera R. (2004). Targeting the mitogen-activated protein kinase cascade to treat cancer. Nat. Rev. Cancer.

[47] Labib K., Tercero J.A., Diffley J.F.X. (2000). Uninterrupted MCM2-7 Function Required for DNA Replication Fork Progression. Science.

[48] Ren B., Yu G., Tseng G.C., Cieply K., Gavel T., Nelson J., Michalopoulos G., Yu Y., Luo J. (2006). MCM7 amplification and overexpression are associated with prostate cancer progression. Oncogene.

[49] Yang J.-Y., Li D., Zhang Y., Guan B.-X., Gao P., Zhou X.-C., Zhou C.-J. (2018). The expression of MCM7 is a useful biomarker in the early diagnostic of gastric cancer. Pathol. Oncol. Res..

[50] Liu Y.-Z., Jiang Y.-Y., Hao J.-J., Lu S.-S., Zhang T.-T., Shang L., Cao J., Song X., Wang B.-S., Cai Y. (2012). Prognostic significance of MCM7 expression in the bronchial brushings of patients with non-small cell lung cancer (NSCLC). Lung Cancer.

[51] Fujioka S., Shomori K., Nishihara K., Yamaga K., Nosaka K., Araki K., Haruki T., Taniguchi Y., Nakamura H., Ito H. (2009). Expression of minichromosome maintenance 7 (MCM7) in small lung adenocarcinomas (pT1): Prognostic implication. Lung Cancer.

[52] Sreenivasan S., Thirumalai K., Krishnakumar S. (2012). Expression Profile of Genes Regulated by Curcumin in Y79 Retinoblastoma Cells. Nutr. Cancer.

[53] Drechsel D., Hyman A.A., Cobb M.H., Kirschner M.W. (1992). Modulation of the dynamic instability of tubulin assembly by the microtubule-associated protein tau. Mol. Biol. Cell.

[54] Wang C., Liu Y., Guo W., Zhu X., Ahuja N., Fu T. (2019). MAPT promoter CpG island hypermethylation is associated with poor prognosis in patients with stage II colorectal cancer. Cancer Manag. Res..

[55] Koo D.-H., Lee H.J., Ahn J.-H., Yoon D.H., Kim S.-B., Gong G., Son B.H., Ahn S.H., Jung K.H. (2015). Tau and PTEN status as predictive markers for response to trastuzumab and paclitaxel in patients with HER2-positive breast cancer. Tumor Biol..

[56] Mimori K., Sadanaga N., Yoshikawa Y., Ishikawa K., Hashimoto M., Tanaka F., Sasaki A., Inoue H., Sugimachi K., Mori M. (2006). Reduced tau expression in gastric cancer can identify candidates for successful Paclitaxel treatment. Br. J. Cancer.

[57] Sekino Y., Han X., Babasaki T., Goto K., Inoue S., Hayashi T., Teishima J., Shiota M., Takeshima Y., Yasui W. (2020). Microtubule-Associated Protein Tau (MAPT) Promotes Bicalutamide Resistance and Is Associated with Survival in Prostate Cancer.

[58] Massague J. (1990). The Transforming Growth Factor-beta Family. Annu. Rev. Cell Biol..

[59] Liao H., Liang Y., Kang L., Xiao Y., Yu T., Wan R. (2021). miR-454-3p inhibits non-small cell lung cancer cell proliferation and metastasis by targeting TGFB2. Oncol. Rep..

[60] Dumont N., Arteaga C.L. (2000). Transforming growth factor-beta and breast cancer: Tumor promoting effects of transforming growth factor-β. Breast Cancer Res..

[61] Lu R., Ji Z., Li X., Qin J., Cui G., Chen J., Zhai Q., Zhao C., Zhang W., Yu Z. (2015). Tumor suppressive microRNA-200a inhibits renal cell carcinoma development by directly targeting TGFB2. Tumor Biol..

[62] Adams J.C. (1997). Thrombospondin-1. Int. J. Biochem. Cell Biol..

[63] Byrne G.J., Hayden K.E., McDowell G., Lang H., Kirwan C.C., Tetlow L., Kumar S., Bundred N.J. (2007). Angiogenic characteristics of circulating and tumoural thrombospondin-1 in breast cancer. Int. J. Oncol..

[64] Albo D., Rothman V.L., Roberts D.D., Tuszynski G.P. (2000). Tumour cell thrombospondin-1 regulates tumour cell adhesion and invasion through the urokinase plasminogen activator receptor. Br. J. Cancer.

[65] Zhang J., Dong M., Li L., Fan Y., Pathre P., Dong J., Lou D., Wells J.M., Olivares-Villagómez D., Van Kaer L. (2003). Endonuclease G is required for early embryogenesis and normal apoptosis in mice. Proc. Natl. Acad. Sci. USA.

[66] Basnakian A.G., Apostolov E.O., Yin X., Abiri S.O., Stewart A.G., Singh A.B., Shah S.V. (2006). Endonuclease G promotes cell death of non-invasive human breast cancer cells. Exp. Cell Res..

[67] Wang X., Tryndyak V., Apostolov E.O., Yin X., Shah S.V., Pogribny I.P., Basnakian A.G. (2008). Sensitivity of human prostate cancer cells to chemotherapeutic drugs depends on EndoG expression regulated by promoter methylation. Cancer Lett..

[68] Dutto I., Tillhon M., Cazzalini O., Stivala L.A., Prosperi E. (2015). Biology of the cell cycle inhibitor p21CDKN1A: Molecular mechanisms and relevance in chemical toxicology. Arch. Toxicol..

[69] Mitsuuchi Y., Johnson S.W., Selvakumaran M., Williams S.J., Hamilton T.C., Testa J.R. (2000). The Phosphatidylinositol 3-Kinase/AKT Signal Transduction Pathway Plays a Critical Role in the Expression of p21WAF1/CIP1/SDI1 Induced by Cisplatin and Paclitaxel. Cancer Res..

[70] Abbas T., Dutta A. (2009). p21 in cancer: Intricate networks and multiple activities. Nat. Rev. Cancer.

[71] Löhr K., Möritz C., Contente A., Dobbelstein M. (2003). p21/CDKN1A mediates negative regulation of transcription by p53. J. Biol. Chem..

[72] Fukazawa T., Guo M., Ishida N., Yamatsuji T., Takaoka M., Yokota E., Haisa M., Miyake N., Ikeda T., Okui T. (2016). SOX2 suppresses CDKN1A to sustain growth of lung squamous cell carcinoma. Sci. Rep..

[73] Liu J., Hu Y., Hu W., Xie X., Ela Bella A., Fu J., Rao D. (2012). Expression and prognostic relevance of p21WAF1 in stage III esophageal squamous cell carcinoma. Dis. Esophagus.

[74] Wei C.-Y., Tan Q.-X., Zhu X., Qin Q.-H., Zhu F.-B., Mo Q.-G., Yang W.-P. (2015). Expression of CDKN1A/p21 and TGFBR2 in breast cancer and their prognostic significance. Int. J. Clin. Exp. Pathol..

[75] Kuang Y.-F., Chen Y.-H. (2004). Induction of apoptosis in a non-small cell human lung cancer cell line by isothiocyanates is associated with P53 and P21. Food Chem. Toxicol..

[76] Zhou Y., Ho W.S. (2014). Combination of liquiritin, isoliquiritin and isoliquirigenin induce apoptotic cell death through upregulating p53 and p21 in the A549 non-small cell lung cancer cells. Oncol. Rep..

[77] Zeng Y., Shen Z., Gu W., Wu M. (2018). Inhibition of hepatocellular carcinoma tumorigenesis by curcumin may be associated with CDKN1A and CTGF. Gene.

[78] Tamura R.E., de Vasconcellos J.F., Sarkar D., Libermann T.A., Fisher P.B., Zerbini L.F. (2012). GADD45 Proteins: Central Players in Tumorigenesis. Curr. Mol. Med..

[79] Ying J., Srivastava G., Hsieh W.-S., Gao Z., Murray P., Liao S.-K., Ambinder R., Tao Q. (2005). The Stress-Responsive Gene GADD45G Is a Functional Tumor Suppressor, with Its Response to Environmental Stresses Frequently Disrupted Epigenetically in Multiple Tumors. Clin. Cancer Res..

[80] Zhang W., Li T., Shao Y., Zhang C., Wu Q., Yang H., Zhang J., Guan M., Yu B., Wan J. (2010). Semi-quantitative detection of GADD45-gamma methylation levels in gastric, colorectal and pancreatic cancers using methylation-sensitive high-resolution melting analysis. J. Cancer Res. Clin. Oncol..

[81] Guo W., Zhu T., Dong Z., Cui L., Zhang M., Kuang G. (2012). Decreased expression and aberrant methylation of Gadd45G is associated with tumor progression and poor prognosis in esophageal squamous cell carcinoma. Clin. Exp. Metastasis.

[82] Yang P., Yuan W., He J., Wang J., Yu L., Jin X., Hu Y., Liao M., Chen Z., Zhang Y. (2009). Overexpression of EphA2, MMP-9, and MVD-CD34 in hepatocellular carcinoma: Implications for tumor progression and prognosis. Hepatol. Res..

[83] Yuan W., Chen Z., Chen Z., Wu S., Guo J., Ge J., Yang P., Huang J. (2012). Silencing of EphA2 inhibits invasion of human gastric cancer SGC-7901 cells in vitro and in vivo. Neoplasma.

[84] Chen L.-X., He Y.-J., Zhao S.-Z., Wu J.-G., Wang J.-T., Zhu L.-M., Lin T.-T., Sun B.-C., Li X.-R. (2011). Inhibition of tumor growth and vasculogenic mimicry by curcumin through down-regulation of the EphA2/PI3K/MMP pathway in a murine choroidal melanoma model. Cancer Biol. Ther..

[85] Moreno-Layseca P., Streuli C.H. (2014). Signalling pathways linking integrins with cell cycle progression. Matrix Biol..

[86] Stewart R.L., O’Connor K.L. (2015). Clinical significance of the integrin α6β4 in human malignancies. Lab. Investig..

[87] Boelens M.C., van den Berg A., Vogelzang I., Wesseling J., Postma D.S., Timens W., Groen H.J.M. (2007). Differential expression and distribution of epithelial adhesion molecules in non-small cell lung cancer and normal bronchus. J. Clin. Pathol..

[88] Chung J., Bachelder R.E., Lipscomb E.A., Shaw L.M., Mercurio A.M. (2002). Integrin (α6β4) regulation of eIF-4E activity and VEGF translation: A survival mechanism for carcinoma cells. J. Cell Biol..

[89] Ni H., Dydensborg A.B., Herring F.E., Basora N., Gagné D., Vachon P.H., Beaulieu J.-F. (2005). Upregulation of a functional form of the β4 integrin subunit in colorectal cancers correlates with c-Myc expression. Oncogene.

[90] Nikolopoulos S.N., Blaikie P., Yoshioka T., Guo W., Giancotti F.G. (2004). Integrin β4 signaling promotes tumor angiogenesis. Cancer Cell.

[91] Khoshnoodi J., Pedchenko V., Hudson B.G. (2008). Mammalian collagen IV. Microsc. Res. Tech..

[92] Burnier J.V., Wang N., Michel R.P., Hassanain M., Li S., Lu Y., Metrakos P., Antecka E., Burnier M.N., Ponton A. (2011). Type IV collagen-initiated signals provide survival and growth cues required for liver metastasis. Oncogene.

[93] Xiao Q., Jiang Y., Liu Q., Yue J., Liu C., Zhao X., Qiao Y., Ji H., Chen J., Ge G. (2015). Minor Type IV Collagen α5 Chain Promotes Cancer Progression through Discoidin Domain Receptor-1. PLoS Genet..

[94] Salomonsson A., Jönsson M., Isaksson S., Karlsson A., Jönsson P., Gaber A., Bendahl P.-O., Johansson L., Brunnström H., Jirström K. (2013). Histological specificity of alterations and expression of KIT and KITLG in non-small cell lung carcinoma. Genes Chromosomes Cancer.

[95] Vliagoftis H., Worobec A.S., Metcalfe D.D. (1997). The protooncogene c-kit and c-kit ligand in human disease. J. Allergy Clin. Immunol..

[96] Krasagakis K., Krüger-Krasagakis S., Eberle J., Tsatsakis A., Tosca A.D., Stathopoulos E.N. (2009). Co-Expression of KIT Receptor and Its Ligand Stem Cell Factor in Merkel Cell Carcinoma. Dermatology.

[97] Reichardt L.F. (2006). Neurotrophin-regulated signalling pathways. Philos. Trans. R. Soc. B Biol. Sci..

[98] Truzzi F., Marconi A., Lotti R., Dallaglio K., French L.E., Hempstead B.L., Pincelli C. (2008). Neurotrophins and Their Receptors Stimulate Melanoma Cell Proliferation and Migration. J. Investig. Dermatol..

[99] Marchetti D., Aucoin R., Blust J., Murry B., Greiter-Wilke A. (2004). p75 neurotrophin receptor functions as a survival receptor in brain-metastatic melanoma cells. J. Cell. Biochem..

[100] Rocha A.S., Risberg B., Magalhães J., Trovisco V., de Castro I.V., Lazarovici P., Soares P., Davidson B., Sobrinho-Simões M. (2006). The p75 neurotrophin receptor is widely expressed in conventional papillary thyroid carcinoma. Hum. Pathol..

[101] Kettunen E., Anttila S., Seppänen J.K., Karjalainen A., Edgren H., Lindström I., Salovaara R., Nissén A.-M., Salo J., Mattson K. (2004). Differentially expressed genes in nonsmall cell lung cancer: Expression profiling of cancer-related genes in squamous cell lung cancer. Cancer Genet. Cytogenet..

[102] Yuanlong H., Haifeng J., Xiaoyin Z., Jialin S., Jie L., Li Y., Huahong X., Jiugang S., Yanglin P., Kaichun W. (2008). The inhibitory effect of p75 neurotrophin receptor on growth of human hepatocellular carcinoma cells. Cancer Lett..

[103] Khwaja F., Tabassum A., Allen J., Djakiew D. (2006). The p75NTR tumor suppressor induces cell cycle arrest facilitating caspase mediated apoptosis in prostate tumor cells. Biochem. Biophys. Res. Commun..

[104] Jin H., Pan Y., Zhao L., Zhai H., Li X., Sun L., He L., Chen Y., Hong L., Du Y. (2007). p75 Neurotrophin Receptor Suppresses the Proliferation of Human Gastric Cancer Cells. Neoplasia.

[105] Tabassum A., Khwaja F., Djakiew D. (2003). The p75NTR tumor suppressor induces caspase-mediated apoptosis in bladder tumor cells. Int. J. Cancer.

[106] Ramsay R.G., Gonda T.J. (2008). MYB function in normal and cancer cells. Nat. Rev. Cancer.

[107] Biroccio A., Benassi B., D’Agnano I., D’Angelo C., Buglioni S., Mottolese M., Ricciotti A., Citro G., Cosimelli M., Ramsay R.G. (2001). c-Myb and Bcl-x overexpression predicts poor prognosis in colorectal cancer: Clinical and experimental findings. Am. J. Pathol..

[108] Greco C., Alvino S., Buglioni S., Assisi D., Lapenta R., Grassi A., Stigliano V., Mottolese M., Casale V. (2001). Activation of c-MYC and c-MYB proto-oncogenes is associated with decreased apoptosis in tumor colon progression. Anticancer Res..

[109] van de Vijver M.J., He Y.D., van’t Veer L.J., Dai H., Hart A.A.M., Voskuil D.W., Schreiber G.J., Peterse J.L., Roberts C., Marton M.J. (2002). A Gene-Expression Signature as a Predictor of Survival in Breast Cancer. N. Eng. J. Med..

[110] Guérin M., Sheng Z.M., Andrieu N., Riou G. (1990). Strong association between c-myb and oestrogen-receptor expression in human breast cancer. Oncogene.

[111] Anfossi G., Gewirtz A.M., Calabretta B. (1989). An oligomer complementary to c-myb-encoded mRNA inhibits proliferation of human myeloid leukemia cell lines. Proc. Natl. Acad. Sci. USA.

[112] Lutwyche J.K., Keough R.A., Hughes T.P., Gonda T.J. (2001). Mutation screening of the c-MYB negative regulatory domain in acute and chronic myeloid leukaemia. Br. J. Haematol..

[113] Cuenda A., Rousseau S. (2007). p38 MAP-Kinases pathway regulation, function and role in human diseases. Biochim. Biophys. Acta Mol. Cell Res..

[114] Liu J., Han L., Li B., Yang J., Huen M.S.Y., Pan X., Tsao S.W., Cheung A.L.M. (2014). F-Box Only Protein 31 (FBXO31) Negatively Regulates p38 Mitogen-activated Protein Kinase (MAPK) Signaling by Mediating Lysine 48-linked Ubiquitination and Degradation of Mitogen-activated Protein Kinase Kinase 6 (MKK6). J. Biol. Chem..

[115] Lotan T.L., Lyon M., Huo D., Taxy J.B., Brendler C., Foster B.A., Stadler W., Rinker-Schaeffer C.W. (2007). Up-regulation of MKK4, MKK6 and MKK7 during prostate cancer progression: An important role for SAPK signalling in prostatic neoplasia. J. Pathol..

[116] Parray A.A., Baba R.A., Bhat H.F., Wani L., Mokhdomi T.A., Mushtaq U., Bhat S.S., Kirmani D., Kuchay S., Wani M.M. (2014). MKK6 is Upregulated in Human Esophageal, Stomach, and Colon Cancers. Cancer Investig..

[117] Lin S., Liu K., Zhang Y., Jiang M., Lu R., Folts C.J., Gao X., Noble M.D., Zhao T., Zhou Z. (2018). Pharmacological targeting of p38 MAP-Kinase 6 (MAP2K6) inhibits the growth of esophageal adenocarcinoma. Cell. Signal..

[118] Satyal S.H., Chen D., Fox S.G., Kramer J.M., Morimoto R.I. (1998). Negative regulation of the heat shock transcriptional response by HSBP1. Genes Dev..

[119] Eroglu B., Min J.-N., Zhang Y., Szurek E., Moskophidis D., Eroglu A., Mivechi N.F. (2014). An essential role for heat shock transcription factor binding protein 1 (HSBP1) during early embryonic development. Dev. Biol..

[120] Shen L., Zhang R., Sun Y., Wang X., Deng A.M., Bi L. (2014). Overexpression of HSBP1 is associated with resistance to radiotherapy in oral squamous epithelial carcinoma. Med. Oncol..

[121] Phan N.N., Wang C.Y., Chen C.F., Sun Z., Lai M.D., Lin Y.C. (2017). Voltage-gated calcium channels: Novel targets for cancer therapy. Oncol. Lett..

[122] Warnier M., Roudbaraki M., Derouiche S., Delcourt P., Bokhobza A., Prevarskaya N., Mariot P. (2015). CACNA2D2 promotes tumorigenesis by stimulating cell proliferation and angiogenesis. Oncogene.

[123] da Costa Prando É., Cavalli L.R., Rainho C. (2011). Evidence of epigenetic regulation of the tumor suppressor gene cluster flanking RASSF1 in breast cancer cell lines. Epigenetics.

[124] Zhang Y., Wang H., Wang J., Bao L., Wang L., Huo J., Wang X. (2015). Global analysis of chromosome 1 genes among patients with lung adenocarcinoma, squamous carcinoma, large-cell carcinoma, small-cell carcinoma, or non-cancer. Cancer Metastasis Rev..

[125] Natrajan R., Little S.E., Reis-Filho J.S., Hing L., Messahel B., Grundy P.E., Dome J.S., Schneider T., Vujanic G.M., Pritchard-Jones K. (2006). Amplification and Overexpression of CACNA1E Correlates with Relapse in Favorable Histology Wilms’ Tumors. Clin. Cancer Res..

[126] Camps M., Nichols A., Arkinstall S. (2000). Dual specificity phosphatases: A gene family for control of MAP kinase function. FASEB J..

[127] Wu G.S. (2007). Role of mitogen-activated protein kinase phosphatases (MKPs) in cancer. Cancer Metastasis Rev..

[128] Keyse S.M. (2008). Dual-specificity MAP kinase phosphatases (MKPs) and cancer. Cancer Metastasis Rev..

[129] Ratsada P., Hijiya N., Hidano S., Tsukamoto Y., Nakada C., Uchida T., Kobayashi T., Moriyama M. (2020). DUSP4 is involved in the enhanced proliferation and survival of DUSP4-overexpressing cancer cells. Biochem. Biophys. Res. Commun..

[130] Li W., Song L., Ritchie A.-M., Melton D.W. (2012). Increased levels of DUSP6 phosphatase stimulate tumourigenesis in a molecularly distinct melanoma subtype. Pigment Cell Melanoma Res..

[131] Bell L.A., Ryan K.M. (2004). Life and death decisions by E2F-1. Cell Death Differ..

[132] Gorgoulis V.G., Zacharatos P., Mariatos G., Kotsinas A., Bouda M., Kletsas D., Asimacopoulos P.J., Agnantis N., Kittas C., Papavassiliou A.G. (2002). Transcription factor E2F-1 acts as a growth-promoting factor and is associated with adverse prognosis in non-small cell lung carcinomas. J. Pathol..

[133] Eymin B., Gazzeri S., Brambilla C., Brambilla E. (2001). Distinct pattern of E2F1 expression in human lung tumours: E2F1 is upregulated in small cell lung carcinoma. Oncogene.

[134] Zhang S.Y., Liu S.C., Al-Saleem L.F., Holloran D., Babb J., Guo X., Klein-Szanto A.J.P. (2000). E2F-1: A Proliferative Marker of Breast Neoplasia. Cancer Epidemiol. Biomark. Prev..

[135] Saiz A.D., Olvera M., Rezk S., Florentine B.A., McCourty A., Brynes R.K. (2002). Immunohistochemical expression of cyclin D1, E2F-1, and Ki-67 in benign and malignant thyroid lesions. J. Pathol..

[136] Huang C.L., Liu D., Nakano J., Yokomise H., Ueno M., Kadota K., Wada H. (2007). E2F1 Overexpression Correlates with Thymidylate Synthase and Survivin Gene Expressions and Tumor Proliferation in Non–Small-Cell Lung Cancer. Clin. Cancer Res..

[137] Coutte L., Dreyer C., Sablin M.-P., Faivre S., Raymond E. (2012). PI3K-AKT-mTOR pathway and cancer. Bull. Cancer.

[138] García Z., Kumar A., Marqués M., Cortés I., Carrera A.C. (2006). Phosphoinositide 3-kinase controls early and late events in mammalian cell division. EMBO J..

[139] Cortés I., Sánchez-Ruíz J., Zuluaga S., Calvanese V., Marqués M., Hernández C., Rivera T., Kremer L., González-García A., Carrera A.C. (2012). p85β phosphoinositide 3-kinase subunit regulates tumor progression. Proc. Natl. Acad. Sci. USA.

[140] Guo Y., Bao Y., Ma M., Zhang S., Zhang Y., Yuan M., Liu B., Yang Y., Cui W., Ansong E. (2017). Clinical significance of the correlation between PLCE 1 and PRKCA in esophageal inflammation and esophageal carcinoma. Oncotarget.

[141] Rosenberg S., Simeonova I., Bielle F., Verreault M., Bance B., Le Roux I., Daniau M., Nadaradjane A., Gleize V., Paris S. (2018). A recurrent point mutation in PRKCA is a hallmark of chordoid gliomas. Nat. Commun..

[142] Arora S., Ranade A.R., Tran N.L., Nasser S., Sridhar S., Korn R.L., Ross J.T.D., Dhruv H., Foss K.M., Sibenaller Z. (2011). MicroRNA-328 is associated with (non-small) cell lung cancer (NSCLC) brain metastasis and mediates NSCLC migration. Int. J. Cancer.

[143] Jiang H., Fu Q., Song X., Ge C., Li R., Li Z., Zeng B., Li C., Wang Y., Xue Y. (2019). HDGF and PRKCA upregulation is associated with a poor prognosis in patients with lung adenocarcinoma. Oncol. Lett..

[144] Nakamura T., Mizuno S. (2010). The discovery of Hepatocyte Growth Factor (HGF) and its significance for cell biology, life sciences and clinical medicine. Proc. Jpn. Acad. Ser. B.

[145] Morishita R., Aoki M., Hashiya N., Yamasaki K., Kurinami H., Shimizu S., Makino H., Takesya Y., Azuma J., Ogihara T. (2004). Therapeutic Angiogenesis using Hepatocyte Growth Factor (HGF). Curr. Gene Ther..

[146] Cipriani N.A., Abidoye O.O., Vokes E., Salgia R. (2009). MET as a target for treatment of chest tumors. Lung Cancer.

[147] Comoglio P.M., Boccaccio C. (2001). Scatter factors and invasive growth. Semin. Cancer Biol..

[148] Ma P.C., Jagadeeswaran R., Jagadeesh S., Tretiakova M.S., Nallasura V., Fox E.A., Hansen M., Schaefer E., Naoki K., Lader A. (2005). Functional Expression and Mutations of c-Met and Its Therapeutic Inhibition with SU11274 and Small Interfering RNA in Non–Small Cell Lung Cancer. Cancer Res..

[149] Ma P.C., Tretiakova M.S., Nallasura V., Jagadeeswaran R., Husain A.N., Salgia R. (2007). Downstream signalling and specific inhibition of c-MET/HGF pathway in small cell lung cancer: Implications for tumour invasion. Br. J. Cancer.

[150] Cecchi F., Rabe D.C., Bottaro D.P. (2012). Targeting the HGF/Met signaling pathway in cancer therapy. Expert Opin. Ther. Targets.

[151] Jiao D., Wang J., Lu W., Tang X., Chen J., Mou H., Chen Q.-Y. (2016). Curcumin inhibited HGF-induced EMT and angiogenesis through regulating c-Met dependent PI3K/Akt/mTOR signaling pathways in lung cancer. Mol. Ther. Oncolytics.

[152] Fischer H.N., Stenling R., Rubio C., Lindblom A. (2001). Colorectal carcinogenesis is associated with stromal expression of COL11A1 and COL5A2. Carcinogenesis.

[153] Vargas A.C., Reed A.E.M., Waddell N., Lane A., Reid L.E., Smart C.E., Cocciardi S., da Silva L., Song S., Chenevix-Trench G. (2012). Gene expression profiling of tumour epithelial and stromal compartments during breast cancer progression. Breast Cancer Res. Treat..

[154] Zeng X.-T., Liu X.-P., Liu T.-Z., Wang X.-H. (2018). The clinical significance of COL5A2 in patients with bladder cancer: A retrospective analysis of bladder cancer gene expression data. Medicine.

[155] Wu D., Chen K., Bai Y., Zhu X., Chen Z., Wang C., Zhao Y., Li M. (2014). Screening of diagnostic markers for osteosarcoma. Mol. Med. Rep..

[156] Bao L., Zhang Y., Wang J., Wang H., Dong N., Su X., Xu M., Wang X. (2016). Variations of chromosome 2 gene expressions among patients with lung cancer or non-cancer. Cell Biol. Toxicol..

[157] Barczyk M., Carracedo S., Gullberg D. (2009). Integrins. Cell Tissue Res..

[158] Jiao Y., Li Y., Liu S., Chen Q., Liu Y. (2019). ITGA3 serves as a diagnostic and prognostic biomarker for pancreatic cancer. OncoTargets Ther..

[159] Tang X.-R., Wen X., He Q.-M., Li Y.-Q., Ren X.-Y., Yang X.-J., Zhang J., Wang Y.-Q., Ma J., Liu N. (2018). MicroRNA-101 inhibits invasion and angiogenesis through targeting ITGA3 and its systemic delivery inhibits lung metastasis in nasopharyngeal carcinoma. Cell Death Dis..

[160] Kurozumi A., Goto Y., Matsushita R., Fukumoto I., Kato M., Nishikawa R., Sakamoto S., Enokida H., Nakagawa M., Ichikawa T. (2016). Tumor-suppressive micro RNA-223 inhibits cancer cell migration and invasion by targeting ITGA 3/ITGB 1 signaling in prostate cancer. Cancer Sci..

[161] Sa K.-D., Zhang X., Li X.-F., Gu Z.-P., Yang A.-G., Zhang R., Li J.-P., Sun J.-Y. (2018). A miR-124/ITGA3 axis contributes to colorectal cancer metastasis by regulating anoikis susceptibility. Biochem. Biophys. Res. Commun..

[162] Sakaguchi T., Yoshino H., Yonemori M., Miyamoto K., Sugita S., Matsushita R., Itesako T., Tatarano S., Nakagawa M., Enokida H. (2017). Regulation of ITGA3 by the dual-stranded microRNA-199 family as a potential prognostic marker in bladder cancer. Br. J. Cancer.

[163] Li H., Wu H., Zhang H., Li Y., Li S., Hou Q., Wu S., Yang S.-Y. (2017). Identification of curcumin-inhibited extracellular matrix receptors in non–small cell lung cancer A549 cells by RNA sequencing. Tumor Biol..

[164] Smrcka A.V. (2013). Molecular targeting of Gα and Gβγ subunits: A potential approach for cancer therapeutics. Trends Pharmacol. Sci..

[165] Khan S.M., Sleno R., Gora S., Zylbergold P., Laverdure J.-P., Labbé J.-C., Miller G.J., Hébert T.E. (2013). The expanding roles of Gβγ subunits in G protein–coupled receptor signaling and drug action. Pharmacol. Rev..

[166] Tanaka H., Kanda M., Miwa T., Umeda S., Sawaki K., Tanaka C., Kobayashi D., Hayashi M., Yamada S., Nakayama G. (2021). G-protein subunit gamma-4 expression has potential for detection, prediction and therapeutic targeting in liver metastasis of gastric cancer. Br. J. Cancer.

[167] Liang L., Zeng J.-H., Qin X.-G., Chen J.-Q., Luo D.-Z., Chen G. (2018). Distinguishable prognostic signatures of left-and right-sided colon cancer: A study based on sequencing data. Cell. Physiol. Biochem..

[168] Katoh M., Nakagama H. (2014). FGF receptors: Cancer biology and therapeutics. Med. Res. Rev..

[169] Li L., Zhang S., Wei L., Wang Z., Ma W., Liu F., Qian Y. (2018). FGF2 and FGFR2 in patients with idiopathic pulmonary fibrosis and lung cancer. Oncol. Lett..

[170] Hosoya T., Nakata A., Yamasaki F., Abas F., Shaari K., Lajis N.H., Morita H. (2012). Curcumin-like diarylpentanoid analogues as melanogenesis inhibitors. J. Nat. Med..

